# A Jurassic record encodes an analogous Dansgaard–Oeschger climate periodicity

**DOI:** 10.1038/s41598-022-05716-8

**Published:** 2022-02-04

**Authors:** Slah Boulila, Bruno Galbrun, Silvia Gardin, Pierre Pellenard

**Affiliations:** 1 CNRS, Institut Des Sciences de La Terre Paris, ISTeP, Sorbonne Université, 75005 Paris, France; 2 ASD/IMCCE, CNRS-UMR8028, Observatoire de Paris, PSL University, Sorbonne Université, 77 Avenue Denfert-Rochereau, 75014 Paris, France; 3 MNHN, CNRS, Centre de Recherche Sur La Paléobiodiversité Et Les Paléoenvironnements, CR2P, Sorbonne Université, 75005 Paris, France; 4 UMR 6282, Biogéosciences, uB/CNRS, Université Bourgogne Franche-Comté, 6 Boulevard Gabriel, 21000 Dijon, France

**Keywords:** Stratigraphy, Sedimentology

## Abstract

Earth’s past climate exhibits short-term (1500-year) pronounced fluctuations during the last glacial period, called Dansgaard–Oeschger (DO) glacial events, which have never been detected in pre-Quaternary times. The record of DO equivalent climate variability in Mesozoic strata can provide constraints on understanding these events. Here we highlight a prominent 1500-year cyclicity in a Jurassic (~ 155 Ma) ice-free sedimentary record from the Tethyan Basin. This Jurassic 1500-year cyclicity is encoded in high-resolution magnetic susceptibility (MS) proxy data reflecting detrital variations, and expressed as marl-limestone couplets. Additionally, MS data detect the modulation of these DO-scale couplets by supercouplet sets, reflecting the precession and its harmonics. We suggest that this Jurassic DO-like cyclicity may originate from paleo-monsoon-like system, analogous to the record of DO events in the Pleistocene East Asian monsoon archives. Paleogeographic reconstructions and atmosphere–ocean simulations further support the potential existence of strong, ancient monsoon circulations in the Tethyan Basin during the Jurassic.

## Introduction

Earth’s past climate exhibits a prominent millennial-scale quasi-cyclic (period of about 1500 years) variability, the origin of which is intensively debated. Such cyclicity, known as the Dansgaard–Oeschger (DO) glacial variations, is characterized by brief, decadal-scale shifts from stadial cold to interstadial warm climates, called the DO events, followed by slow transitions back to stadials within centuries to millennia^1^. Single DO events have been simulated to result from atmospheric temperature warming of up to 15 °C within a few decades, pointing to abrupt and severe changes in Earth’s past climate^1,2^. The driving mechanisms of DO events have received increasing attention in recent years because of their global record in continental and marine environments, especially in glacial archives of the last glacial period. Ice-core records from the Greenland, and deep-sea sediment records from the North Atlantic Ocean highlight these prominent DO glacial events. Paleoclimatic studies have now shown that the 1500-year climate cycle is no longer restricted to the North Atlantic Ocean of the last glacial period^3^. The 1500-year cycle is documented in both hemispheres, in other oceans and in continents, such as in lake and river deposits^4–6^, in pollen fossils^7^, in stalagmite proxy records^8^, and in loess–paleosol deposits^3^.

**Figure 1:**
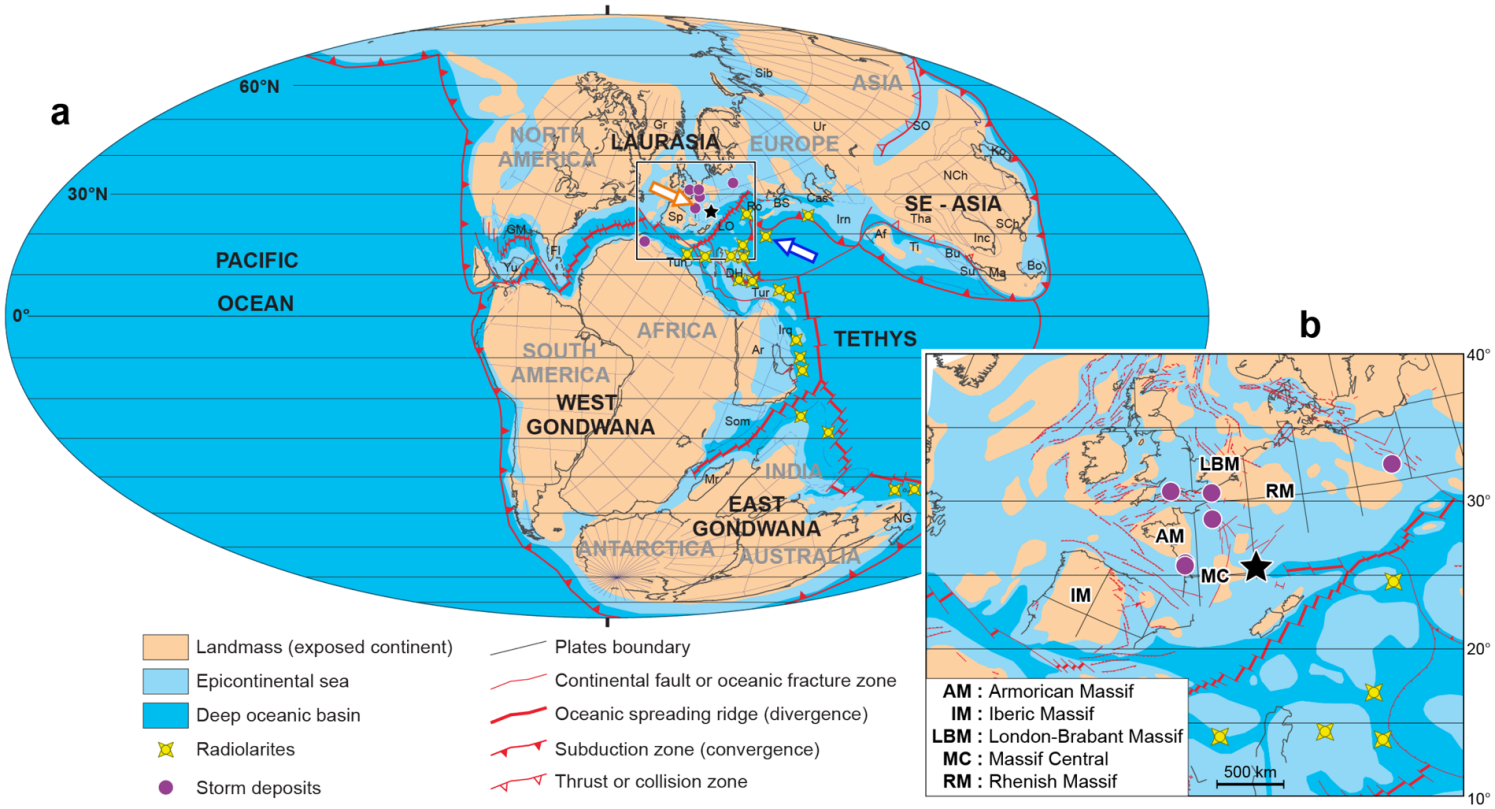
Late Jurassic (Kimmeridgian) paleogeographic Earth’s reconstruction. **(a)** Global map^85^. The Subalpine Basin is indicated by a black star. Potential paleomonsoon-like system showing atmospheric circulations in the Northwestern Tethyan Ocean. Northern Hemisphere summer (orange arrow) and winter (blue arrow). **(b)** A focus on the northwestern Tethyan realm (modified from Thierry^86^^75^^81–84,87–90^). Sites recording Kimmeridgian radiolarities are from Cecca et al. Sites recording Kimmeridgian storm deposits are compiled for the present study.

**Figure 2:**
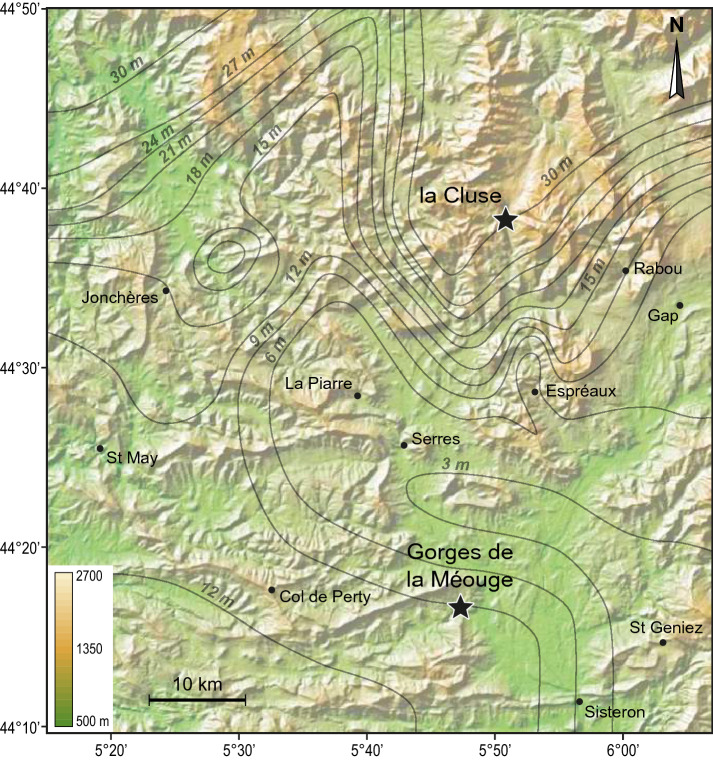
Topographic map of the studied La Cluse site, and other sites recording the Lothari ammonite Subzone (Early Kimmeridgian) in southeastern France, and isopachs of the Lothari ammonite Subzone (modified from Pederneiras ^24^). This figure was generated by Adobe Illustrator CS6 software (https://helpx.adobe.com/fr/illustrator/using/links-info.html). Stratigraphic correlation of the La Cluse section with its time-equivalent La Méouge (indicated by stars) is shown in the Supplementary Figure S8.

The detection of DO analogous periodicity in pre-Quaternary sedimentary records can further support its global nature. In particular, the record of DO equivalent periodicity in ice-free geological epochs can provide insights into the primary exciting force of DO events, and its possible independency to ice-sheet dynamics. Here we explore millennial-scale climate variability in a greatly expanded, cyclic sedimentary succession of a greenhouse (ice-free) Jurassic period. The studied section, called the La Cluse, was deposited in the epicontinental marine Subalpine Basin (Tethyan Ocean, Fig. 1), and is now exposed in southeastern France ( Figs. 1 and 2, Supplementary figure S1). The La Cluse section is characterized by an exceptionally high sedimentation rate with respect to time-equivalent sections in the Subalpine Basin (Fig. 2), hence prone to detect high-frequency climate variability. Using ultra-highly resolved magnetic susceptibility sediment data at La Cluse we find a prominent millennial-scale cyclicity of a period close to 1500 years (henceforth 1.5 kyr, 1 kyr = 1000 years), modulated by the precession cycle and its harmonics.

## Results and interpretation

### Cyclostratigraphic analysis of the MS dataset

**Figure 3:**
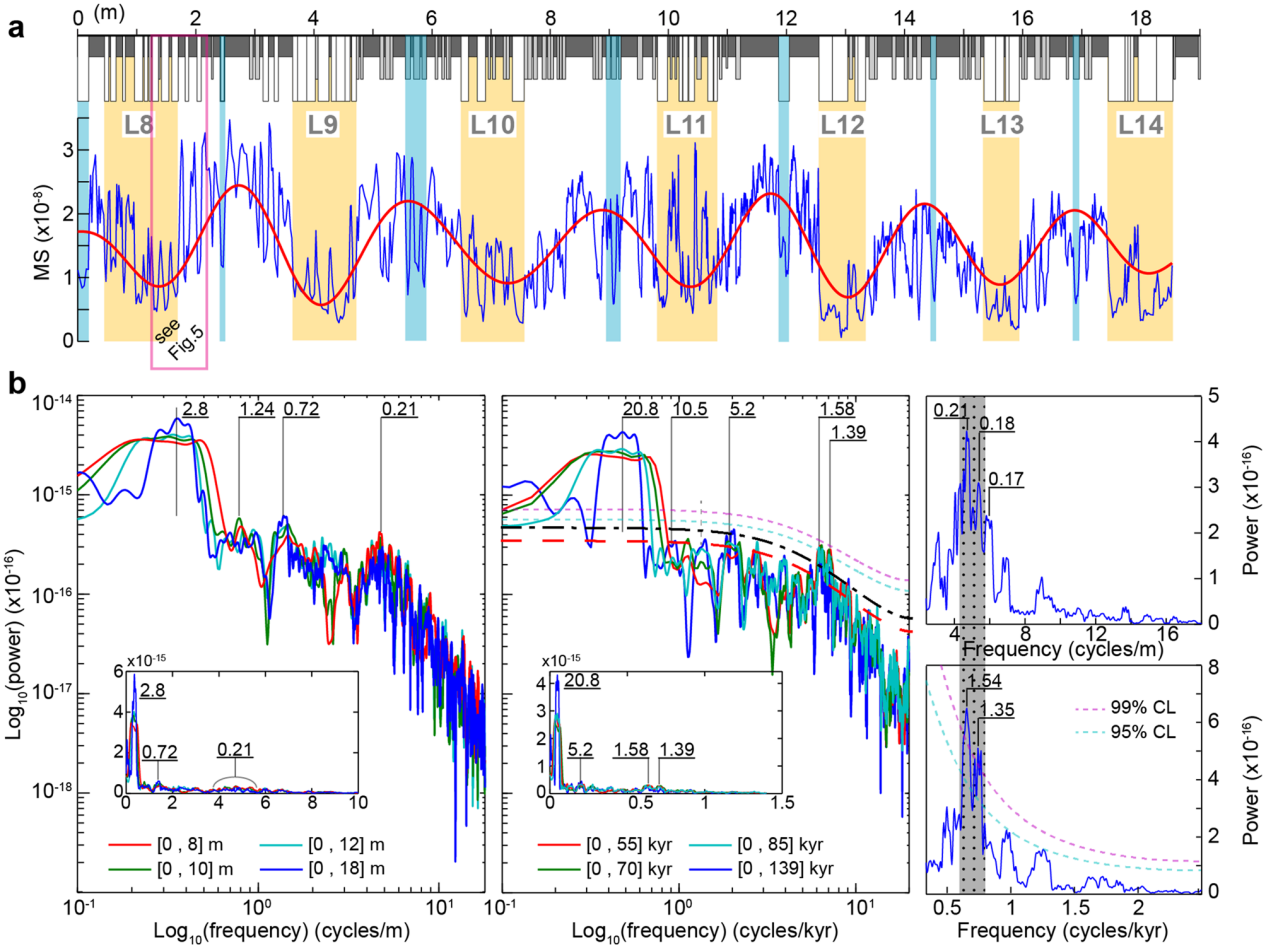
Lithostratigraphy and magnetic susceptibility (MS) data of the Lothari ammonite Subzone *p.p.* (Early Kimmeridgian, Late Jurassic, ~ 155 Ma) at La Cluse sedimentary succession (Subalpine Basin, SE France), and spectral analysis of MS data. **(a)** Lithostratigraphy and MS data, along with bandpass (2.8 m) filtering of the precession MS related cycles and basin-wide correlated precession cycle extremes L8 through L14 lithostratigraphic markers, which correspond to carbonate-rich intervals. For lithology, white: limestone, light grey: marly limestone, and dark grey: marls. **(b)** 2pi-MTM power spectra. Left panel: spectra per intervals in the stratigraphic (m) domain, middle panel: spectra per intervals in the time (kyr) domain (see “Methods” and Fig. 4), right panel: spectra of the interval 0–8 m and its equivalent in the time domain with a focus on the 1.5 kyr cycle band. *Insets* in the left and middle panels are the same spectra but in a linear scale and with tuncated Nyquist frequencies at 10 and 1.5 cycles/m (instead of 18 and 2.5 cycles/m respectively). Grey-dashed area in the right panel indicates the two close peaks at the beating frequencies of the 1.5 kyr cycle band that generate the half-precession periodicity. Remarkably, the interval from the base of the section till L10 shows a strong modulation of the 1.5 kyr cycle by the half precessional cycle (illustrated in Supplementary figures S5–S7). This is ultimately related to the high sedimentation rate within this interval that enhances the record of the 1.5 kyr cycle (see Fig. 4f).

**Figure 4:**
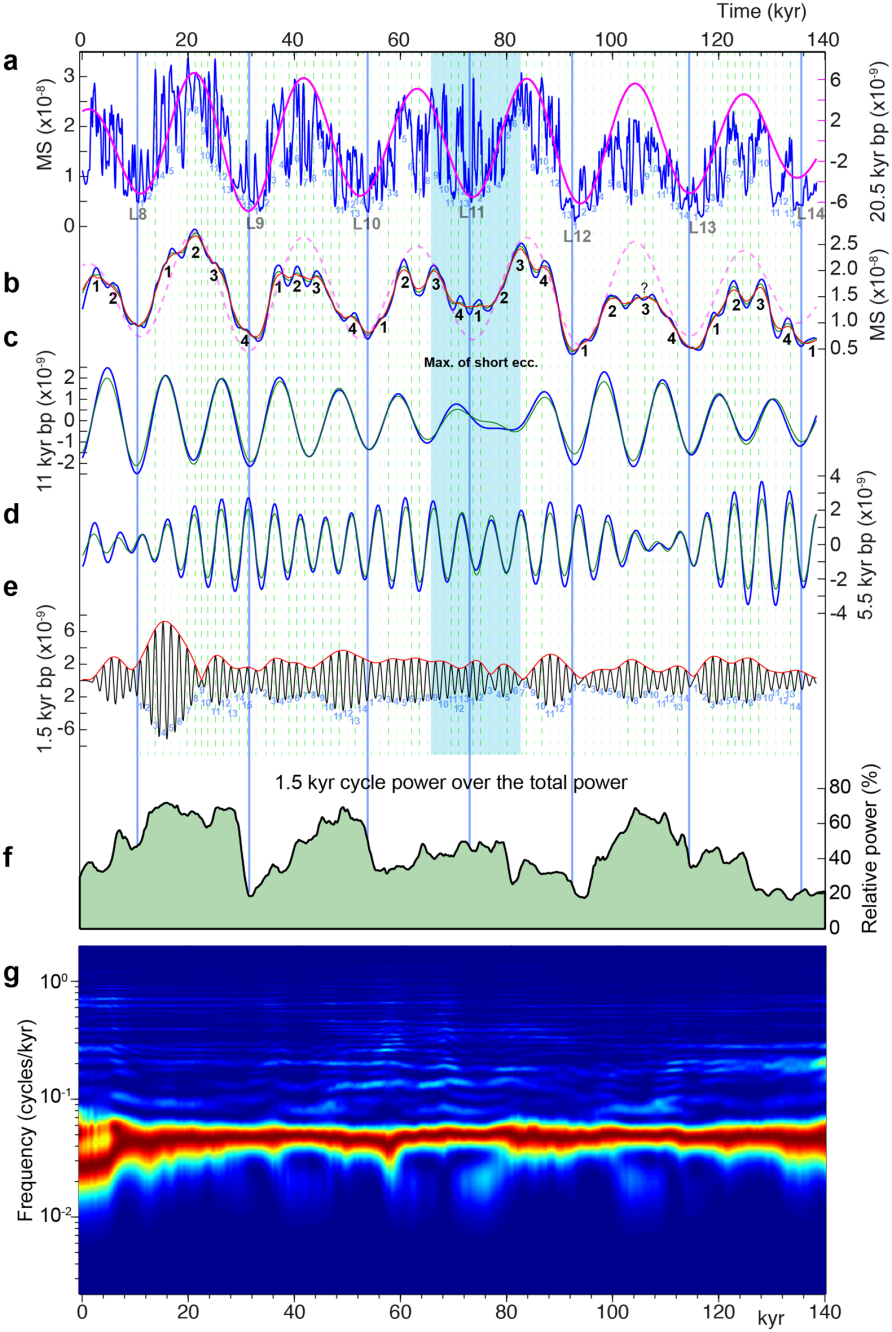
Time-series analysis of the tuned MS data. (**a**) Tuned MS data. The 1.5 kyr cycles, which correspond to the highest frequency cyclicity (marl-limestone couplets), are numbered within each precession cycle, from 1 to 13 or 14. L8 through L14 are as in Fig. 3. **(b)** SSA first RC1 component indicates precession and its harmonics (half- and quarter-precession). Quarter-precession cycles within each precession cycle are numbered from 1 to 4. **(c)** Bandpass filtered half-precession cycles from the raw tuned MS. **(d)** Bandpass filtered quarter-precession cycles from the raw tuned MS. **(e)** Bandpass filtered 1.5 kyr cycle band along with Hilbert amplitude modulation (AM) envelopes. Light blue-shaded area, within L11 lithostratigraphic marker, indicates a maximum of short eccentricity cycling (see Supplementary figure S8). **(f)** Evolutive MTM power spectra to highlight the power of the 1.5 kyr cycle relative to the total power of MS signal along the time series. The cutoff frequencies are 0.4 and 1.2 cycles/kyr. **(g)** Evolutive FFT spectrogram using 50 kyr window and 0.2 kyr step, normalized to the strongest amplitude in order to highlight the precession cycle. The evolutive FFT spectrograms in the stratigraphic domain are shown in the Supplementary figure S9.

We generated a highly resolved (~ 130 year) 139 kyr-long Late Jurassic magnetic susceptibility (MS) sediment record on the basis of ultra-high resolution sampling (~ 1 to ~ 2 cm) of a ~ 18.5 m thick marl-limestone succession (see “Methods” and Supplementary Information), at the La Cluse section, that exceptionally crops out in the Subalpine Basin of southeastern France (Fig. 1). MS paleoclimate proxy data encode rhythmic lithologies, composed of calcareous-rich (limestone) beds alternating with marly layers (Fig. 3). These rhythmic lithologies were orbitally paced by the precession modulated by Earth’s orbital eccentricity^9–13^, thus reflecting climatically driven *in-situ* carbonate production versus detrital flux into the Subalpine Basin in a Late Jurassic greenhouse climate^14^. Spectral analysis of the highly resolved MS data (Figs. 3 and 4) detects a strong climatic precession signal, and in particular two close cyclicities of ~ 1.3 and ~ 1.5 kyr matching the smallest, elementary marl-limestone cyclicity (Figs. 3 and 4e, Supplementary figures S2-S7). Each precession cycle contains twelve to fifteen elementary marl-limestone couplets (Fig. 4a,e and Supplementary figures S3-S5). Interestingly, amplitude modulation (AM) analysis of these two close cyclicities indicates that they are strongly modulated by the precession and its harmonics (Fig. 4 and Supplementary figures S2-S7). Field observation reveals a striking modulation of the elementary marl-limestone couplets by supercouplet sets matching especially the precession and its first (half) harmonic (Supplementary figures S2-S5). Thus, our results suggest that the ~ 1.5 kyr sedimentary cyclicity, is modulated by the precession and its harmonics. Then, we tentatively associated such striking ~ 1.5 kyr Late Jurassic sedimentary cyclicity to the Quaternary DO climate variability, which has also a periodicity at around 1.5 kyr^6,7^. The impact of decrease in sedimentation rate on the record of the 1.5 kyr cycle was assessed at the precession-scale cycle (Fig. 4f,g and Supplementary figure S9). Detailed field description does not show any sedimentological structure that may reflect potential hiatuses. This is supported by the continuous spectral line depicting the precession wavelength (Supplementary figure S9).

To further investigate the modulation of DO analogous periodicity by the climatic precession in this Jurassic record, we test the potential effect of tidal dissipation on drifting the linked DO/precession periodicities^15,16^. We have then applied a set of tuning based on DO equivalent periodicity (from 1.30 to 1.80 kyr with an increment of 50 years) to look for the calibrated precession cycles, and compare them with the predicted present and Late Jurassic periodicities^15,16^. Results indicate that tuning wavelength of elementary marl-limestone couplets to a mean period of 1.45 kyr calibrates the precession wavelengths to periods of 21.43 and 17.71 kyr, matching the predicted Jurassic precession values (Supplementary figures S8 and S10). Tuning to a mean period of 1.55 kyr (Supplementary figure S10) calibrates the precession-scale cycles to periods of 22.93 and 18.83 kyr respectively. Such calibrated precession cycles are much longer than those predicted for the Jurassic time, but very close to those predicted for the present time (Supplementary figure S10). Thus, this linkage in the shortened DO and precession periods further argues the above modulation of DO equivalent cycles by the precession.

### Potential impact of diagenesis on marl-limestone couplets and MS signal

**Figure 5:**
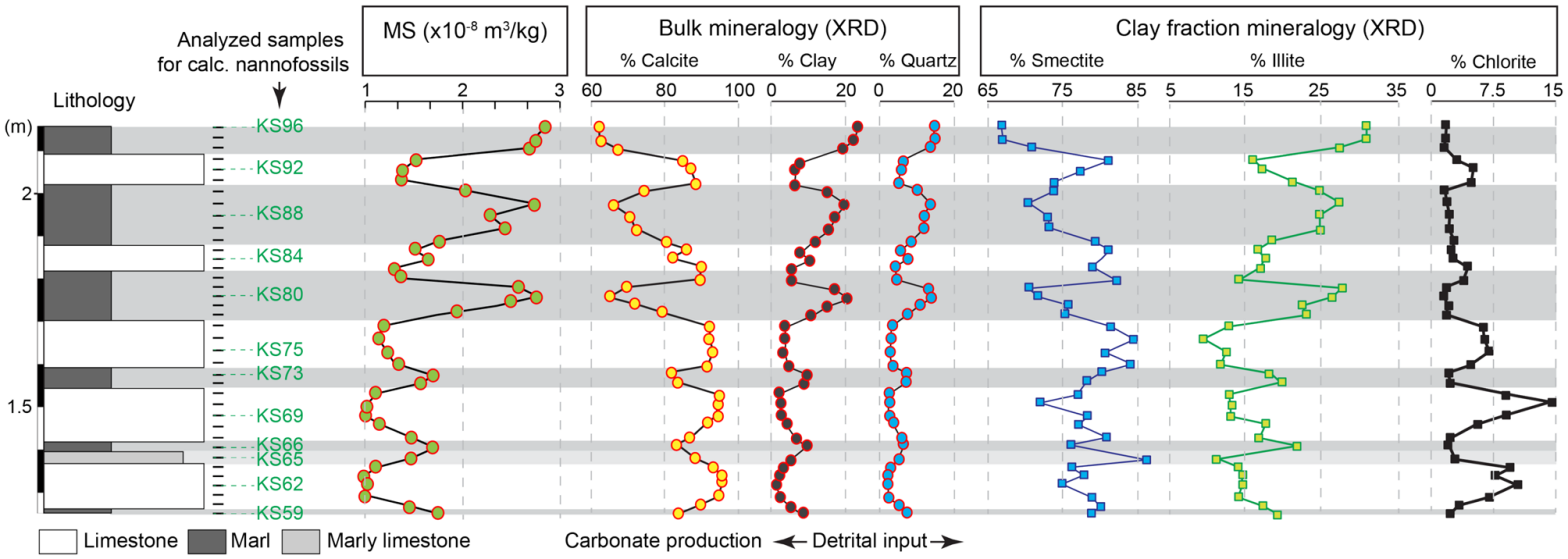
Integrated magnetic susceptibility (MS), bulk and clay fraction mineralogy in five successive marl-limestone couplets at La Cluse section showing similar cyclic variations between MS, detrital component (quartz and clay) and detrital illite in marly interbeds, reflecting enhanced continental runoff conditions. Numbers in green depict the samples used for calcareous nannofossil analyses.

**Figure 6:**
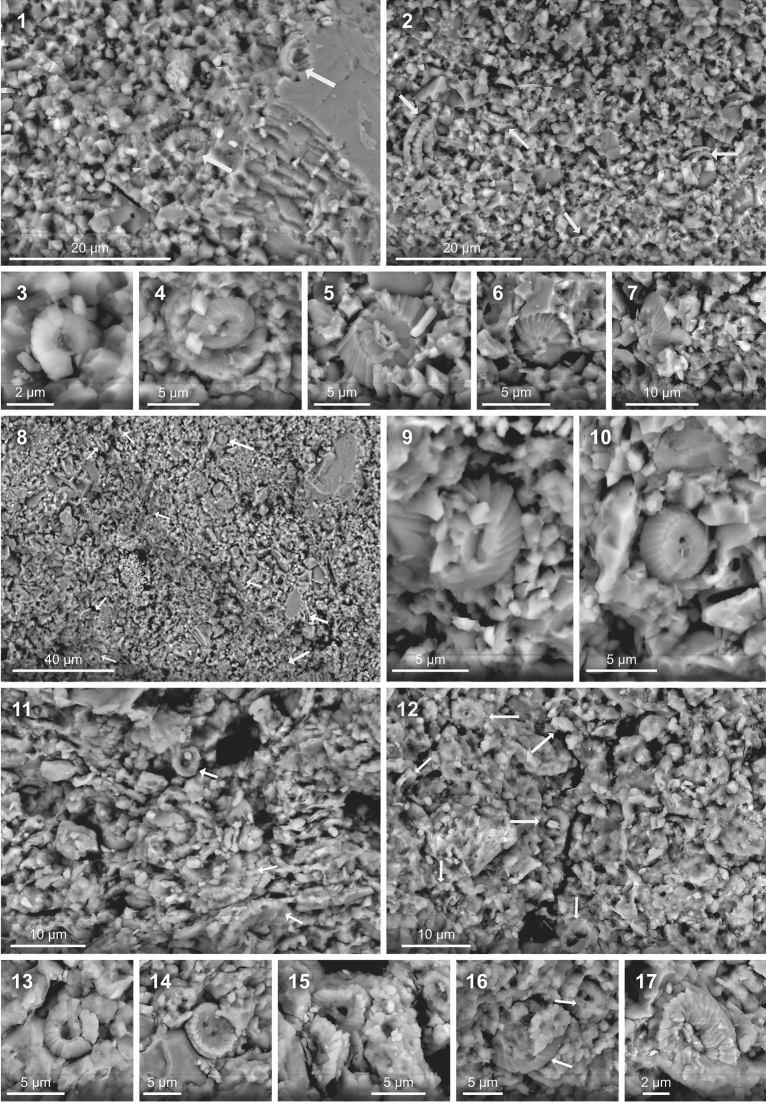
Scanning Electron Microscope (SEM) micrographs of nannofacies. See Fig. 5 for the stratigraphic position of each sample. Scale bar is reported on each micrograph. 1 and 2: Freshly broken limestone surface (samples KS62 and KS69 respectively). Calcareous nannofossils as fabric component are well visible (arrows show only a part of these components) as moulds (1) and fragments (2). 3: *Biscutum* sp (sample KS62). 4: Placoliths of *Watznaueria* (sample KS84). 5: Coccolith of *Watznaueria* distal view (sample KS62). 6: Coccolith of *Watznaueria* as mould (sample KS92). 7: Coccolith of *Watznaueri* side view (sample KS69). 8: Freshly broken surfaces of marly limestone (sample KS65). 9 and 10: Close-up of coccoliths indicated by arrows in (8). 11 and 12: Freshly broken surface of marls (samples KS73 and KS80 respectively). Calcareous nannofossils as fabric component are present as entire and fragmented coccoliths (arrows). 13: *Discorhabdus* sp (sample KS80). 14: Proximal view of coccolith *Watznaueria britannica* (sample KS80). 15 and 17: Coccoliths of *Watznaueria* (sample KS80).

In order to investigate the composition of marls and limestones and to check the preservation of the primary environmental signal in the MS dataset, we performed calcareous nannofossil and mineralogical analyses (see “Methods”) on five DO-scale cycles (i.e., five marl-limestone couplets) (Fig. 5). Scanning Electron Microscope (SEM) observations of nannofacies of the marls and limestones show that the limestones consist of fine-grained micritic mudstones, and highlight the omnipresence of coccoliths at different stages of preservation, fragmentation and molds in both marl and limestone fabrics (Fig. 6). We did not perform a quantitative study of nannofossil carbonate flux because it was not the principal aim of the work. However, our visual observations of the micritic limestones (Fig. 6) point to the remarkable occurrence of coccolith debris as a principal fabric component of carbonate.

Preservation might vary within a same sample, where well and bad preserved coccoliths can co-exist. The most recognisable coccoliths in the limestones are of a variable size (0.5 to 8 μm) and belong to genera *Watznaueria*, *Cyclagelosphaera,* placoliths of robust structure and resistant to diagenenis, although some *Biscutum* and *Stephanolithion* genera, known to be more fragile and dissolution prone, occur too (Fig. 6). Marly lithologies contain a greater number of coccoliths than limestone beds because preservation of nannofossils can be enhanced by the presence of clay that, to some extent, exerts a protective function and allows easier isolation of individual coccoliths^17^. The omnipresence of coccoliths and coccolith fragments in both marls and limestones at La Cluse and in other time-equivalent sections in the Subalpine Basin^14,17^ further supports an in situ pelagic, climatically driven carbonate production, mostly by coccoliths.

**Figure 7:**
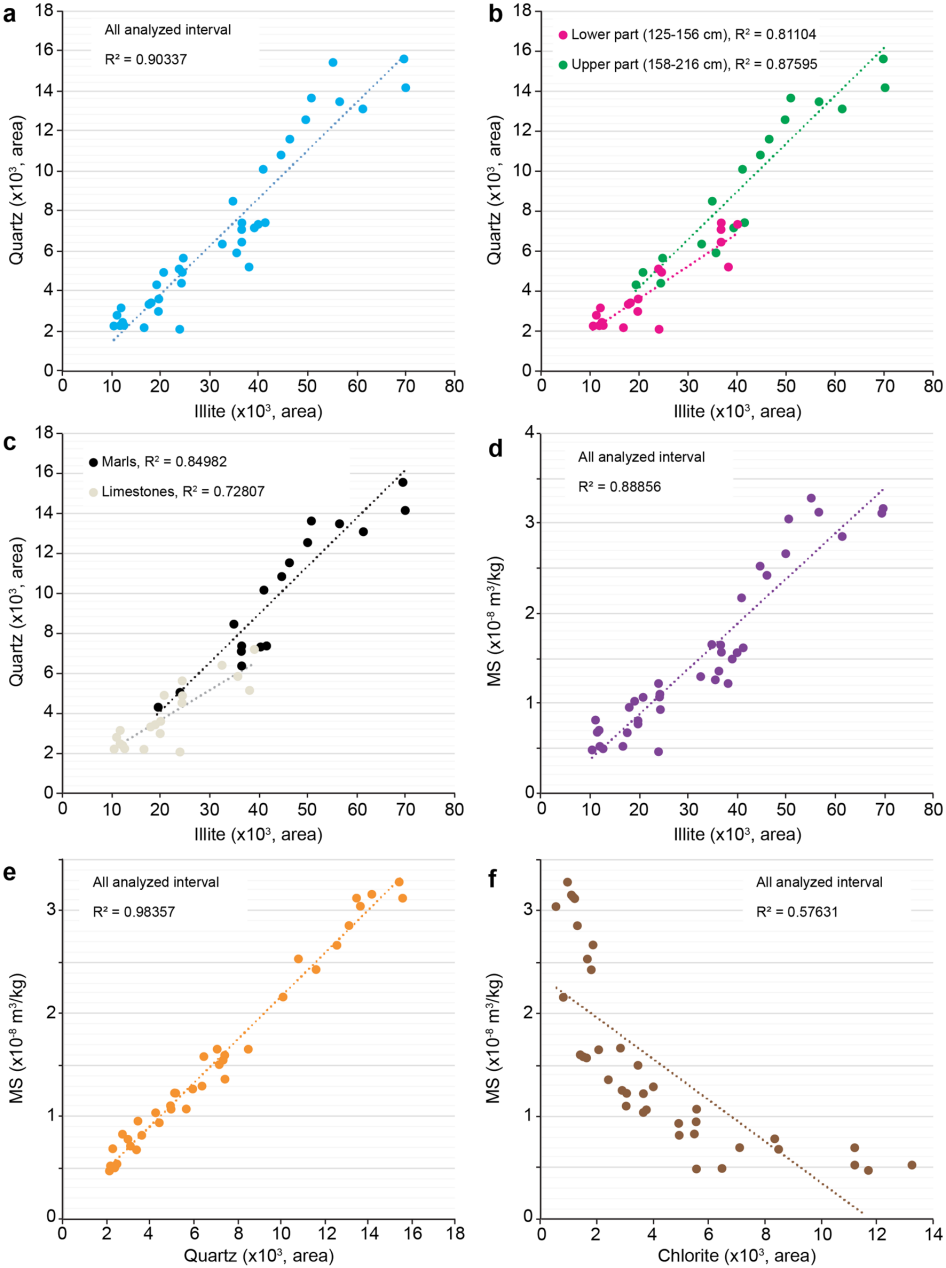
Cross-plots of the multiple studied proxies. Measured values for minerals (clay minerals and quartz) correspond to the main diffraction peak areas **(a)** Illite–Quartz correlation. **(b)** Illite–Quartz correlation over two distinct intervals, 125–156 cm (showing significant variations of chlorite content) and 158–216 cm (showing weak variations of chlorite content). **(c)** Illite–Quartz correlation over the two main lithologies, only limestones (white points) and only marls (black points). **(d)** Illite–magnetic susceptibility (MS) correlation. **(e)** Quartz–MS correlation. **(f)** Chlorite–MS correlation.

Bulk and clay mineralogy analyses provide additional constraints on the preservation of the primary climate signal expressed in the marl-limestone alternations and encoded in the MS dataset. The high smectite content (> 65%) in clay assemblages (Fig. 5) is a clear evidence that the burial diagenesis was limited in this part of the basin. Instead, smectite minerals would result from detrital clays deposited in a semi-arid and/or seasonaly dominated climate at this intertropical latitude during the Late Jurassic^18^ (Fig. 1). In addition, variation in smectite versus illite content, recording the marl-limestone alternations, further support that the lithology was driven by climate change. In particular, the higher content of detrital illite minerals in marls would reflect enhanced precipitation favoring increased runoff on landmasses (Fig. 5). Such a feature is expressed by the strong correlation between the detrital components (clay and quartz contents in the bulk fraction) and illite content in the clay fraction. The MS signal shows similar variations, which are mainly controlled by detrital paramagnetic (e.g. illite) minerals. Such evidence can be supported by the cross-plots of illite-quartz and illite-MS (Fig. 7). These three detrital-input proxies show a strong, positive correlation, especially in the marly intervals. Accordingly, the MS signal very likely reflects a primary detrital-flux origin from enhanced erosional processes linked to changes in humidity/aridity conditions.

However, chlorite content that systematically increases with carbonate content, particularly when limestone beds are thicker (Fig. 5), likely reflect diagenetic effect in carbonate-rich beds as it was previously demonstrated in the Subalpine Basin^19^. This implies that early diagenetic effect cannot be excluded on post-depositional of the limestones. Nevertheless, the low proportions of chlorite, less than 15% (and less than 5% for the upper limestone beds), suggest a very limited early diagenesis, hence chlorite content cannot be responsible for the MS signal as shown in Fig. 7. This is further supported by the strong positive correlation of MS with the illite and clay contents, and not with the chlorite content (Figs. 5 and 7). Finally, the evolution of clay assemblages in this weakly, diagenetically affected marl-limestone alternations suggests contrasting climate conditions linked to the continental runoff as it was previously demonstrated in other Mesozoic marl-limestone successions^20^.

In summary, calcareous nannofossil and clay mineralogy analyses rule out a severe diagenesis, and hint at a potential early diagenesis which is not enough strong to obliterate the primary environmental signal^14,17,21^, encoded in the MS dataset. Several studies based on detailed mineralogical, paleontological, and geochemical data suggested that post-depositional diagenesis of marl-limestone couplets may not only distort primary environmental signals, but also mimic primary signals^22,23^. Lateral correlations of these alternating lithologies at the scale of the Subalpine Basin, over many tens of kilometers^9,10,13,24^, exclude a purely local diagenetic origin of marl-limestone alternations from initially homogeneous sediments^25,26^. Additionally, the continuously gradual variations of carbonate content, which closely track the highest frequency cyclicity corresponding to the basic marl–limestone couplets, are typical of a periodic process that controls their deposition. Lower-frequency cyclicity (i.e., supercouplets) modulates the couplets in an orbital precession/eccentricity fashion^10,13^. In particular, in time-equivalent sections, we have shown the potential use of MS in the detection/preservation of the primary Milankovitch climate cycles, also expressed in marl-limestone facies^12,13^ (see also for e.g., Fig. 1 of Boulila et al.^21^). There are numerous studies showing the powerful use of MS as a proxy for Mesozoic cyclostratigraphy^12,13,27,28^. Therefore, our results exclude a severe diagenetic overprint that could obliterate the primary signal, although a very early diagenesis is likely, which could mimic or amplify the primary (climate) marl-limestone lithological signal, expressed in the MS data.

## Discussion

### On the periodic nature of Quaternary DO events and their amplitude modulations

**Figure 8:**
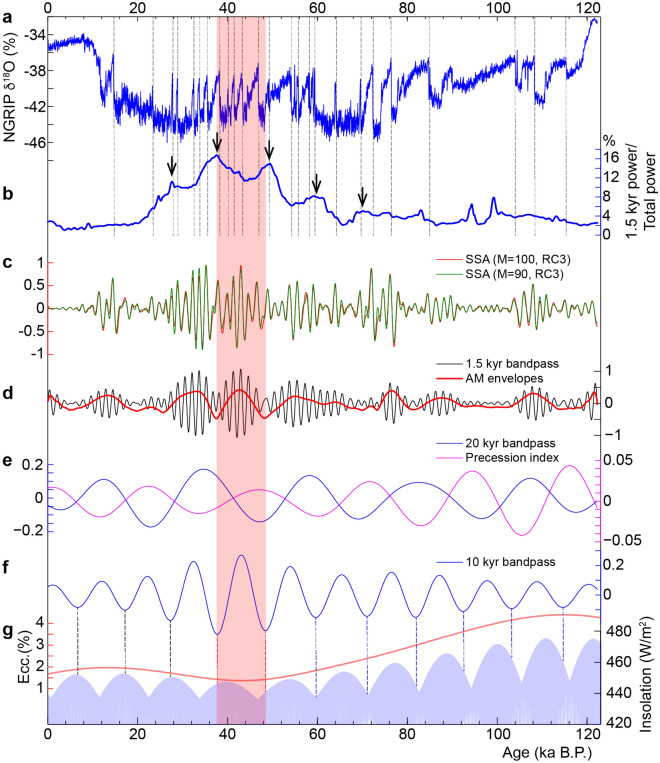
Amplitude modulation (AM) analysis of NGRIP climate proxy data at the 1.5 kyr cycle band. **(a)** δ^18^O record from Greenland ice core obtained from NGRIP at a regular sampling rate of 20 years (see “Methods”). Dansgaard-Oeschger (DO) Events are indicated by vertical dashed lines. **(b)** Evolutive power spectra of the 1.5 kyr cycle band (1.5 kyr power over the total δ^18^O power). Sliding window = 20 kyr, 1.5 kyr selected band = 0.6 ± 0.1 cycles/kyr, MTM time-bandwidth product = 2. Black arrows indicate the half-precession cycles modulating DO events. **(c)** SSA reconstructed components (RC) to isolate the 1.5 kyr cycle band. **(d)** Bandpass flitered δ^18^O data at the 1.5 kyr cycle band (0.6 ± 0.1 cycles/kyr) and Hilbert transform output (AM envelopes). **(e)** 20 kyr bandpass filered (0.043 ± 0.015 cycles/kyr) AM envelopes along with the precession index. **(f)** 10 kyr bandpass filered (0.095 ± 0.02 cycles/kyr) AM envelopes. **(g)** The equatorial insolation modelled with a sampling step of one month (see “Methods”). The light blue-shaded area depicts the annual cycle, which is modulated by the first precession harmonic (half-precession), the precession and the eccentricity cycles (lower insolation values are truncated at almost the mean value of 420 W/m^2^) (the complete data over a longer time interval of 500 ka is shown in Supplementary Fig. S12). The red curve depicts Earth’s orbital eccentricity. Orange-shaded area depicts the change in phase correlation at half-precession band, between astronomical and paleoclimatic variations.

**Figure 9:**
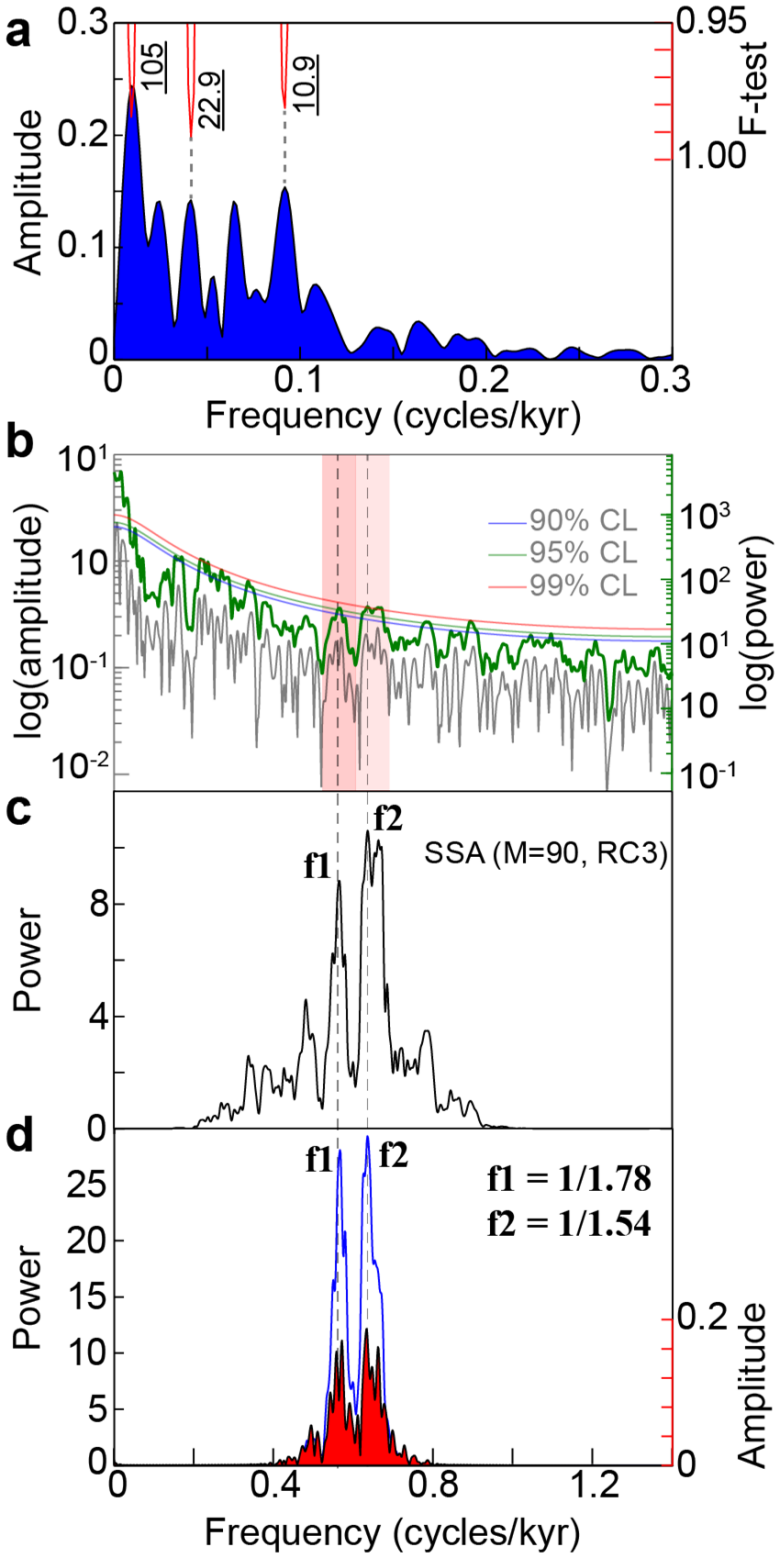
Amplitude and power spectra of NGRIP δ^18^O data. **(a)** Amplitude spectrum of AM of the 1.5 kyr band (red curve in Fig. 8d). **(b)** Amplitude and power spectra of the raw δ^18^O data (curve in Fig. 8a). **(c)** Power spectrum of SSA RC3 (green curve in Fig. 8c). **(d)** Amplitude and power spectra of the 1.5 kyr bandpass filtered δ^18^O data (black curve in Fig. 8d). The vertical dashed lines indicate the two beating frequencies f1 and f2 producing the 10.9 kyr period shown in the upper panel in ‘a’.

Numerous studies argue for or against the cyclic nature of the Quaternary DO glacial events. Deciphering the cyclic vs acyclic character of DO events provides a valuable information on their physical drivers. In general, studies for the cyclic behavior suggest an external driving force^29–32^, while studies against suggest internal factors to the climate system^33,34^. Although we hint at a period centered on ~ 1.5 kyr for the Quaternary DO glacial events^29–32,35^, time-series analysis of updated ages and data of the North Atlantic ice-rafted debris indicates that the 1.5 kyr cycle could potentially arise from an averaging artifact of ~ 1000 and ~ 2000 cycles that are also observed within the Holocene at multiple locations^36^. To account for such DO climate variability, a pseudo-periodicity of ~ 1.5 ± 0.5 kyr was suggested^36^. Furthermore, there are several studies arguing against the periodic nature of DO climate variability or at least against its continuity over the last glacial period^34,36,37^. For instance, Schulz^37^ argued that the ~ 1.5 kyr is limited to only DO events 5, 6, and 7 (between 31 and 36 ka B.P.), while Ditlevsen et al. ^34^ suggested that the waiting times between subsequent DO events are within the high-likelihood range of an exponential distribution, implying that the timing of the DO events is consistent with a Poisson process and should correspond to a statistical artifact. Our analyses of the evolution of Quaternary DO cycicity further support that this cyclicity is not continuously recorded over the last glacial period^37^, and its amplitude was mainly focused on the interval from approximately 24 to 63 ka (Fig. 8a,b). Additionally, our AM analysis of DO cycle band indicates the modulation by the precesssion and its harmonics, especially its first harmonic (10 kyr, Figs. 8c–g, 9 and Supplementary Fig. S14). Interestingly, we noted a switch from in-phase to antiphase relationship at half-precession band within the interval ~ 38–48 ka (Fig. 8f,g). This transitional phase is intriguingly coincident with the interval showing the maximal power of DO events (Fig. 8b). Correlation with Earth’s orbital eccentricity indicates that this transitional phase matches minimal values of eccentricity (Fig. 9g), suggesting that DO events may also be phase-modulated at longer timescale by the eccentricity. In particular, the interval from approximately 24 to 63 ka, which documents the strongest DO events, corresponds to a minimum of orbital eccentricity modulated precession (Fig. 8 and Supplementary Fig. S15). Although we hint at a a potential link between DO cycle band and low-latitude insolation forcing in the Quaternary and the Jurassic records, the modulation of DO-scale band by Milankovitch cycles does not necessarily imply that DO events were paced by cyclic insolation forcing. The presence of both Milankovitch and DO-scale bands in the sedimentary climate records can result from two independent forcing processes, i.e. insolation for Milankovitch band, and internal forcing for DO events^38^. The interfering periods at the 1.5 kyr band that can produce the precession and its harmonics, as observed in the Jurassic record (~ 1.3 vs. ~ 1.5 kyr, Fig. 3) and Quaternary records (1.54 versus 1.78 kyr, Fig. 9 and Supplementary figure S14), can arise from a simple time calibration of DO-scale variability by low-frequency precession (and its harmonics) band in the sedimentary archive. We should also note that the Jurassic record reveals more harmonic variations at the DO-like period band. It is likely that the intrinsec property of ice-sheet dynamics in Quaternary climates, that creates saw-tooth shape in the glacial data proxies, may be responsible for the obliteration of the forcing cyclicity in the glacial archives. However, during ice-free periods such as the Jurassic, the forcing DO equivalent cyclicity would be better preserved. The physical (lithological) expression of the Jurassic DO analogous cyclicity as marl-limestone couplets may support the hypothesis of a periodic forcing process of Quaternary DO events^29–32^.

### Global nature and low-latitude records of DO analogous periodicity

Several studies suggest internal drivers of DO events from periodic calving of the Greenland ice sheet, and oscillations in the atmosphere–ocean system^33,38,39^. The detection of DO equivalent cycles during the last glacial period in terrestrial and marine environments, in both hemispheres and across all latitudes^1,7,40^ points to an external driving force^29,31,32^. It is likely that the DO events were generated via interaction between ice and atmosphere–ocean systems, and that low-latitude insolation may act as a modulator of these events^41^. The record of the ~ 1.5 kyr equivalent climate cycle during the ice-free Late Jurassic epoch further reinforces its global nature, and suggests that the primary exciting force is independent to ice-sheet dynamics. Additionally, the 1.5 kyr signal is not restricted to the last glacial period but is a pervasive feature of Holocene climate variability^7,29,42^. Millennial-scale climate variabilities including the 1.5 kyr periodicity have also been detected in several intervals of the past 1 Ma^3,43^. There are two competing hypotheses for the origin of DO events, whether they originate from low latitudes or from northern high latitudes. The low-latitude generated climate signal would expand earthwide via advective processes^44,45^. Our finding of AM of DO-scale cycle band by the precession and its harmonics and the intertropical paleolatitudinal position of the studied tethyan site hint at a low-latitude origin of this Jurassic signal. The half-precession signal is typical of low-latitude regions, interpreted as a result of the twice-yearly passage of the Sun over the equator^46,47^. At the same time, our finding does not rule out a primary high-latitude signal of Quaternary DO events. It is also likely that this Jurassic DO-like cyclicity does not reflect the Quaternary DO variability, and does not share causal mechanisms. However, it is intriguing that similar Jurassic and Quaternay climatic and oceanographic processes were potentially involved in the record of DO-like cycles.

A strong half-precession signal was detected during the Pleistocene not only in the equatorial Pacific and Atlantic oceans, but also in northeastern Atlantic (~ 50°N), suggesting climate transfer process from lower towards higher latitudes via advective transport^44^. Another evidence for such low-latitude signal and its transfer to the higher latitudes comes from correlation of climate proxy record of half-precession forced equatorial (Atlantic) zonal wind fields, and subpolar millennial-scale ice rafting Heinrich events^41,45^. In the tropical Pacific ocean, half-precession and DO equivalent cyclicities were detected with high fidelity in the same paleoclimate record (0–45 ka) in Australia^48^. Furthermore, model and proxy evidence of DO events shows a global interconnection between the two hemispheres through atmospheric and oceanic circulations along with meridional migrations of the intertropical convergence zone (ITCZ)^49^. The ITCZ, which reflects the interhemispheric heat balance, is more sensitive to low-latitude insolation forcing. Proxy evidences for abrupt changes in tropical circulation and precipitation, synchronous with North Hemisphere DO events, have been derived from sediment cores^50^, and speleothem records^40^.

Given the modern geography at low latitudes, among the fundamental mechanisms involved in orbitally forced atmospheric-continental-oceanic changes are the monsoon and the El Niño–Southern Oscillation (ENSO) systems. These processes, which are very sensitive to the seasonal and annual insolation cycles^51,52^, were very likely active during the Mesozoic^53,54^. Such mechanisms are climate catalysts for the delivery of heat and moisture from low to high latitudes. Indeed, the impact of low-latitude insolation forcing on polar and subpolar climates via the advective transport from these processes has long been suggested^41,44,45,55^. The monsoon system is strongly shaped by large-scale meridional temperature gradients and the related position of the ITCZ^56^. DO-like cycles have been recorded in loess–paleosol sequences and in lake deposits, possibly in relation with fluctuations of the ITCZ^3,5^.

DO equivalent climate variability is well expressed in numerous Late Pleistocene palaeo-monsoon records from all over the globe, along with a strong low-latitude precessional signal^40,57^. Connection between paleo-monsoon and North Atlantic paleoclimate sites has been demonstrated at the mellennial-scale variability^57,58^. Paleoclimate archives of the past 45 ka in the tropical Pacific ocean at paleo-ENSO site register both half-precession and DO equivalent cyclicities suggesting that these cyclicities modulated variation in the ENSO^48^. This is supported by ocean–atmosphere modeling, which emphasizes the role of orbitally driven intertropical regions in pacing ENSO, and potentially DO events^52^. In summary, models and proxies show evidence for low-latitude insolation forced continental and oceanographic mechanisms, and their translated impact to high latitudes.

The Subalpine Basin was an epicontinental sea in the Tethyan domain, located at an intertropical paleolatitude during the Late Jurassic (Fig. 1), an area sensitive to low-latitude orbital forcing. Numerous studies of Mesozoic strata have shown that the marl-limestone alternations in the Subalpine Basin were orbitally paced by low-latitude precession forcing, modulated by the eccentricity^11,12^. In particular, the La Cluse sedimentary succession and its time equivalents have been proven to be driven by precession-eccentricity forcing^10,12,13,59^. There are also some lines of evidence for orbitally modulated paleomonsoon-like system in the Subalpine Basin during the Jurassic and Cretaceous, responsable for the formation of the cyclic sedimentary successions^60,61^. Half-precession cycles have also been documented in other Jurassic Subalpine sections, where high sedimentation rates allow their detection^62^. The common climate feature in the Jurassic and Quaternary records of 1.5 kyr DO equivalent periodicity may be the monsoon system, under the direct influence of the tropical ITCZ^40,63,64^.

### Jurassic Tethyan paleo-monsoon record of DO analogous periodicity

During the Late Jurassic, Pangean continentality was large, and the most important landmasses were the "Laurasia" and "southeast Asia in the north and west, and the "East Gondwana" in the south (Fig. 1a). These extensive continental areas, especially at low- and mid-latitudes, were likely favorable for the development of very active, strong monsoons along the intertropical Tethyan zones^64–66^.

Modelling experiments and stratigraphic studies suggest that the supercontinent Pangea during the Mesozoic was the subject of a megamonsoonal atmospheric circulation that led to extreme seasonality^53,64–70^. The first ocean general circulation model for an idealized Tethyan Ocean^66^ showed intense westward, winter surface winds alternated with intense eastward, summer surface winds at approximately the paleolatitude (20 to 25°N) of the Tethyan Subalpine Basin (Fig. 1). Simulations of Early and Late Jurassic (Kimmeridgian) climate boundary conditions show similar results, along with the dominance of monsoon-like system over the Tethys^67,68,71,72^. In winter, a large high-pressure cell was created between colder Laurasia and warmer Panthalassa, which forced clockwise atmospheric circulation. The resulting winter paleo-trade winds blew westward over the Tethyan Ocean, and were reversed during the summer^66,68^.

Based on calcareous nannofossil and clay mineralogy datasets, together with previous data and models of the Late Jurassic tethyan domain, we propose a depositional model of the clay and carbonate deposits. We suggest that the La Cluse lithological marl-limestone alternations may have been paced by a Late Jurassic Tethyan monsoon system. Clays were likely derived from weathering of the several continental blocks that rimmed the Subalpine Basin (Fig. 1b) during humid, winter monsoon circulations blowing across the open Tethyan Ocean (Fig. 1a). These enhanced runoff conditions are supported by the abondance of illite minerals in the marls by increasing mechanical erosion on landmasses (Fig. 5). Carbonates were potentially in situ produced by calcareous nannofossils^14^ (see Section above) during westerly, dry summer monsoon circulations coming from continental and shallow-marine environments (Fig. 1a), favoring in turn the formation of upwelling cells with higher marine-surface productivity^73^. The drier climatic conditions within the limestone beds are supported by reduced detrital inputs and higher contents of smectites that is prone to be transported on longer distance compared to other clay minerals and formed during semi-arid or seasonally contrasted climate (Fig. 4). Modeling shows that these seasonally alternated monsoon winds may have strong effects on ocean currents, especially in the northern hemisphere^66,68,74^. There are multiple Early Kimmeridgian sedimentological indicators arguing for ocean fertility and intense upwelling systems in the Tethyan domain^70,75^. For instance, radiolarites were common in Early Kimmeridgian time, developed in several, specific tethyan sites between 25N and 20S^75^ (Fig. 1b), and their occurrence may indicate increased fertility in sea surface waters^70,76^. Additionally, these Late Jurassic radiolarites were demonstrated to be paced by Milankovitch orbital forcing^77^. Despite the paleoenvironmental setting of the studied La Cluse section (epicontinental sea) is quite different to those of deep-sea sections recording radiolarites (Fig. 1), the occurrence of radiolarites in relatively near areas during the Early Kimmeridgian could be a good indicator of fertile tethyan seawaters in relation with upwelling cells^70^. Thus, we suggest that Milankovitch forcing can modulate the DO analogous climate cycles in the Subalpine Basin by driving, via the monsoon system, intense continental runoff and the deposition of clays during heavy winter rainfalls, alternated by higher marine carbonate productivity during very active summer upwelling systems.

There is generally good agreement between the model climate and the existing geological data. Kimmeridgian climate simulations show that southern Europe region experienced very dry summers but moist winters^78,79^. This is in support with the reconstructed, westward tropical cyclone pathways over Europe during winter seasons, inferred on the basis of both geological and model data^80^. Such tropical cyclones were potentially intense due to crossing air masses over the large, open Tethyan Ocean in the west^80^. There are a number of Kimmeridgian sedimentary successions in Europe and elsewhere, which show storm deposits in shallow-marine and coastal settings, possibly revealing severe winter storms^81–84^.

## Conclusions

Our highly resolved Late Jurassic record from the Subalpine Basin (southeastern France) documents Dansgaard–Oeschger (DO) analogous periodicity (~ 1500 yrs), expressed in the lithological variations as elementary marl-limestone couplets. These DO-scale couplets are modulated by supercouplet sets, reflecting the precession and its harmonics. The dominance of precession and its harmonics is characteristic of the equatorial and intertropical climate regions. The Subalpine Basin belonged to the intertropical Tethyan sea during the Late Jurassic, likely influenced by low-latitude insolation forced monsoon-like system, which was potentially responsible for the deposition of the cyclic marl-limestone alternations. Therefore, we conclude that the analyzed paleoclimate record of the Late Jurassic further supports the global nature of DO-like events, and in particular that their potential primary cause is independent to ice-sheet dynamics. This Jurassic record of DO equivalent periodicity may be analogous to Late Pleistocene East Asian monsoon archives that also document DO-scale climate variability, due to the specific palaeogeography of the Subalpine Basin and climate conditions in the Late Jurassic.

## Methods

The studied La Cluse section is situated in the Subalpine Basin of southeastern France (Fig. 1 and Supplementary figure S1), in the southern part of the Devoluy mountain Chain (44°38′26.76″N, 5°50′12.43″E), about twenty kilometers of Gap (Fig. 2). The section exceptionally crops out along the Abéou river. It covers a part of the Lothari ammonite Subzone of the Hypselocyclum Zone (Early Kimmeridgian), and is composed of marl-limestone alternations of pelagic facies, as attested by nannofossil analyses. The studied time interval is extremely thick at La Cluse, compared to other time-equivalent sections (Supplementary figure S8). The variation of the thickness of the Lothari ammonite Subzone in the Subalpine Basin was related to a variable paleobathymetry, which was very important at La Cluse site (Fig. 2). The mean paleobathymetry of the Subalpine Basin during the Late Juarrsic was assessed as ranging from several hundreds to few thousands of meters^91^.

We acquire ultra high-resolution (~ 1 to ~ 2 cm) magnetic susceptibility (MS) data at the La Cluse section. A total of 1101 samples were collected, and measured using an AGICO Kappabridge MFK1-B susceptometer. Each sample was measured three times, and the mean of these values is reported after weight normalization. The standard deviation of triplicate measurements is always < 0.0091 × 10^–8^ m^3^/kg. MS data indicate cyclical fluctuations in clay versus carbonate contents from the relative contribution of paramagnetic clay minerals and diamagnetic calcium carbonate minerals (Supplementary figure S8). The lithostratigraphic log (Fig. 3) is obtained from a rough field description, based mainly on the hardness of marly levels versus limestone beds. Therefore, the highly resolved MS dataset, which is an indirect measure of the lithology, provides more details on the lithological variations. Thus, the mismatch between MS and the lithostratigraphic field derived log observed in some intervals of the La Cluse section is due to the rough lithological field description.

To convert the thickness into the time domain, we apply integrated ammonite biostratigraphy and cyclostratigraphy including correlations with other sections in the basin (Supplementary figure S8). The resulting mean temporal resolution of the studied interval is ~ 0.130 kyr. This Jurassic record documents with high fidelity the 1.5 kyr signal, however, in the uppermost part of the section when sedimentation rate decreases it is barely recorded (Figs. 3 and 4). Field description points to a facies dominated by regular marl-limestone alternations, and does not show any sedimentological structure that may reflect potential hiatuses. Change in sedimentation rate along the section was assessed using the evolutive FFT^28^.

We used amplitude and power spectral analyses based on the Thomson’s multitaper method with three windows, together with the harmonic F-test and robust red noise to seek evidence for individual lines within frequency bands of both high and low powers^91^. We perform amplitude modulation (AM) analysis to extract low frequency AM envelopes of the 1.5 kyr cycle band using the Hilbert transform. Prior to AM analysis, bandpass filtering is carried out via the Gaussian filter^28^. Bandpass filtering is applied conjointly with the Singular Spectrum Analysis (SSA) method^91^ to isolate high-frequency bands, with low amplitudes. SSA method separates signals from noise in a sequence of signal components (reconstructed components, RCs, e.g., Fig. 4) that are statistically independent, at zero lag, and based on signal strength (variance). It is an important feature of SSA that the signals can be amplitude and phase modulated. Thus, SSA method was used in a complementary way to AM analysis (i.e., Gauss-Hilbert; e.g., Fig. 4). All used Earth’s orbital parameters are based on La2004 astronomical model^16^.

Precession driven insolation could impact over a large latitudinal band but with relative decreasing intensity towards higher northern latitudes (Supplementary figure S11). However, half-precession induced insolation has preponderant influence on low-latitude regions, as a result of the twice-yearly passage of the Sun over the equator^46,47^. The spectrum of amplitude of the seasonal cycle of the energy that the equatorial and intertropical regions receive from the Sun is focused on the eccentricity and harmonics of the precession. Also, the annual cycle is modulated by Milankovitch parameters, and at low latitudes it is strongly modulated by the eccentricity, precession and harmonics of the precession (Fig. 7 and Supplementary figures S12 and 13). The response of the climate system to larger insolation values in the equatorial and intertropical zones would produce half-precession cycles in these regions^47^ (Supplementary figure S13).

We then applied AM and spectral analyses to climate proxy data of the last glacial period to check AM outputs, as inferred from the Jurassic record. AM results reinforce the hypothesis of modulation of DO-cycle band by the precession and its harmoncis (Figs. 8, Fig. 9 and Supplementary Figure S14). We used coinjointly the highly resolved δ^18^O data from Greenland ice core obtained during NGRIP^28^, at a regular sampling rate of 20 years^92^, and Antarctic δ^18^O data from the WAIS ice core obtained by WAIS Divide Project Members, 2013^93^ using WD2014 chronology^95^. The evolution of δ^18^O amplitudes related to DO events, especially during the last glacial period, was quantified using 2π multi-taper variance (power) spectra^96^, which assesses the ratio of 1.5 kyr cycle variance over the total variance in the signal. This is a complementary technique to assess AM of DO events (see arrows in Fig. 8b).

Finally, we have tested the effect of diagenesis on the formation of marl-limestone alternations at La Cluse. We analyzed five successive marl-limestone couplets using calcareous nannofossil and clay mineralogy approaches. Calcareous nannofossils were investigated by scanning electron microscope (SEM) FlexSem 1000-II, Hitachi. Eleven samples taken from the middle parts of marls and limestones of successive marl-limestone couplets (Fig. 5) were processed for SEM analyses: a chips of fresh sediment was fixed on a carbon stub. We aimed to get a rapid estimate of the potential contribution of nannofossils to CaCO_3_ content, and the potential impact of diagenesis on the marls versus limestones. Clay fraction (< 2 µm) mineralogical bulk mineralogical analyses were performed using X-Ray Diffraction (XRD) on all samples covering the five successive marl-limestone couplets (38 samples) for potential correlations with the MS dataset. For the clay fraction analyses, powdered samples were decarbonated with a 0.2 M HCl solution, then the fraction below two microns was extracted, smeared on oriented glass slides and run in a Brucker D8 Endeavour diffractometer with CuKα radiations, LynxEye detector and Ni filter under 40 kV voltage and 25 mA intensity (GISMO platform, Biogeosciences laboratory, University of Burgundy). Three runs were performed for each sample to discriminate clay phases: 1) Air-drying; 2) Ethylene–glycol solvation; 3) Heating at 490 °C. Clay minerals were identified using their main diffraction (d001) peak and semi-quantificaiton obtained by comparison of peak areas using the MacDiff 4.2.5 software on glycolated diffractograms. The same semi-quantification procedure was applied for the bulk mineralogy using the main peak diffraction of each mineral phase recognized. The proportions of calcite were corrected by additionnal calcium carbonate measurements using a Bernard calcimeter with 3 replicates per measured samples. Uncertainties for both clay fraction and bulk mineralogy analyses are less than 5%.

## Supplementary Information

Supplementary Information 1.

Supplementary Information 2.

Supplementary Information 3.
