# Author Correction: A Jurassic record encodes an analogous Dansgaard–Oeschger climate periodicity

**DOI:** 10.1038/s41598-022-08065-8

**Published:** 2022-03-09

**Authors:** Slah Boulila, Bruno Galbrun, Silvia Gardin, Pierre Pellenard

**Affiliations:** 1 CNRS, Institut Des Sciences de La Terre Paris, ISTeP, Sorbonne Université, 75005 Paris, France; 2 ASD/IMCCE, CNRS-UMR8028, Observatoire de Paris, PSL University, Sorbonne Université, 77 Avenue Denfert-Rochereau, 75014 Paris, France; 3 MNHN, CNRS, Centre de Recherche Sur La Paléobiodiversité Et Les Paléoenvironnements, CR2P, Sorbonne Université, 75005 Paris, France; 4 UMR 6282, Biogéosciences, uB/CNRS, Université Bourgogne Franche-Comté, 6 Boulevard Gabriel, 21000 Dijon, France

Correction to: *Scientific Reports* https://doi.org/10.1038/s41598-022-05716-8, published online 04 February 2022

The original version of this Article contained an error in Affiliation 4, which was incorrectly given as ‘UMR 6282, uB/CNRS, Université Bourgogne Franche-Comté, 6 Boulevard Gabriel, 21000 Dijon, France’. The correct affiliation is listed below:

UMR 6282, Biogéosciences, uB/CNRS, Université Bourgogne Franche-Comté, 6 Boulevard Gabriel, 21000 Dijon, France

In addition, the original version of this Article contained an error in the legend of Figure 4.

“REVISED: Time-series analysis of the tuned MS data.”

now reads:

“Time-series analysis of the tuned MS data.”

The original Article has been corrected.

