# Supplementary material for: A Jurassic record encodes an analogous Dansgaard–Oeschger climate periodicity
Source: Sci Rep. 2022 Feb 4;12:1968. doi: 10.1038/s41598-022-05716-8 (PMC8817006; doi:10.1038/s41598-022-05716-8)
Supplement: Supplementary file 1 — Supplementary Information 1. [file 41598_2022_5716_MOESM1_ESM.doc]

**Supplementary Information on:**

**A Jurassic record encodes an analogous Dansgaard–Oeschger climate periodicity**

**Slah Boulila^a,b,*^, Bruno Galbrun^a^, Silvia Gardin^c^, and Pierre Pellenard^d^**

^a^ Sorbonne Université, CNRS, Institut des Sciences de la Terre Paris, ISTeP, F-75005 Paris, France.

^b^ ASD/IMCCE, CNRS-UMR8028, Observatoire de Paris, PSL University, Sorbonne Université, 77 Avenue Denfert-Rochereau, 75014 Paris, France.

^c^ Sorbonne Université, MNHN, CNRS, Centre de Recherche sur la Paléobiodiversité et les Paléoenvironnements, CR2P, F-75005 Paris, France.

^d^ Biogéosciences, UMR 6282, uB/CNRS, Université Bourgogne Franche-Comté, 6 Boulevard Gabriel, 21000 Dijon, France.

^*^ Corresponding author. Tel: +33.144274163. E-mail: [slah.boulila@sorbonne-universite.fr](mailto:slah.boulila@sorbonne-universite.fr).

^*^ Correspondence. Tel: +33.144274163; Fax: +33.144273831. E-mail: [slah.boulila@sorbonne-universite.fr](mailto:slah.boulila@sorbonne-universite.fr).

The supplementary file includes the following figures and tables:

• *Supplementary figure S1:* Present location of the La Cluse and the La Méouge sections and Paleogeographic framework of the Vocontian Basin.

• *Supplementary figure S2:* Field photograph of the lower part of the studied La Cluse section.

• *Supplementary figure S3:* Field interpretation of the 1.5 kyr cycle, bundled by quarter- and half-precession cycles along with the lithostratigraphic log.

• *Supplementary figure S4:* Field interpretation of the 1.5 kyr cycle, bundled by half-precession and precession cycles.

• *Supplementary figure S5:* Focus on the lower part of the La Cluse section, to show the ratio of 1/12 to 1/15 between the presumed DO analogous and precession periodicities.

• *Supplementary figure S6:* Time-series analysis of magnetic susceptibility (MS) proxy data of the lower part of the La Cluse section.

• *Supplementary figure S7:* Generalized Lomb-Scargle (GLS) periodograms of magnetic susceptibility proxy data of the lower part of the La Cluse section.

• *Supplementary figure S8:* Approach used to convert thickness into time.

• *Supplementary figure S9:* Evolutive FFT spectrograms showing the continuity of the precession spectral line in the stratigraphic domain.

• *Supplementary figure S10:* 1.5 kyr tuning of wavelengths of elementary marl-limestone couplets, and comparison of the calibrated precession periods to the theoretically predicted Jurassic and present values.

• *Supplementary figure S11:* Relative amplitudes of precession and obliquity as expressed in variations of the boreal summer insolation through latitudes.

• *Supplementary figure S12:* Equatorial insolation over the past 500 ka, modelled with a sampling step of one month.

• *Supplementary figure S13:* Potential origin of the half-precession cycles at low latitudes.

• *Supplementary figure S14:* Amplitude modulation (AM) analysis of δ^18^O NGRIP and WAIS data at the 1.5 kyr cycle band.

• *Supplementary figure S15:* Potential amplitude modulation (AM) od DO-scale cycles by the short eccentricity.

• *Supplementary table S1, entitled "MS Data":* The magnetic susceptibility (MS) dataset of the studied La Cluse section (first column: stratigraphic height in meter and the second column: MS values).

• *Supplementary table S2, entitled "ClayMineralogy":* The bulk and clay mineralogy datasets of the selected interval of the La Cluse section.


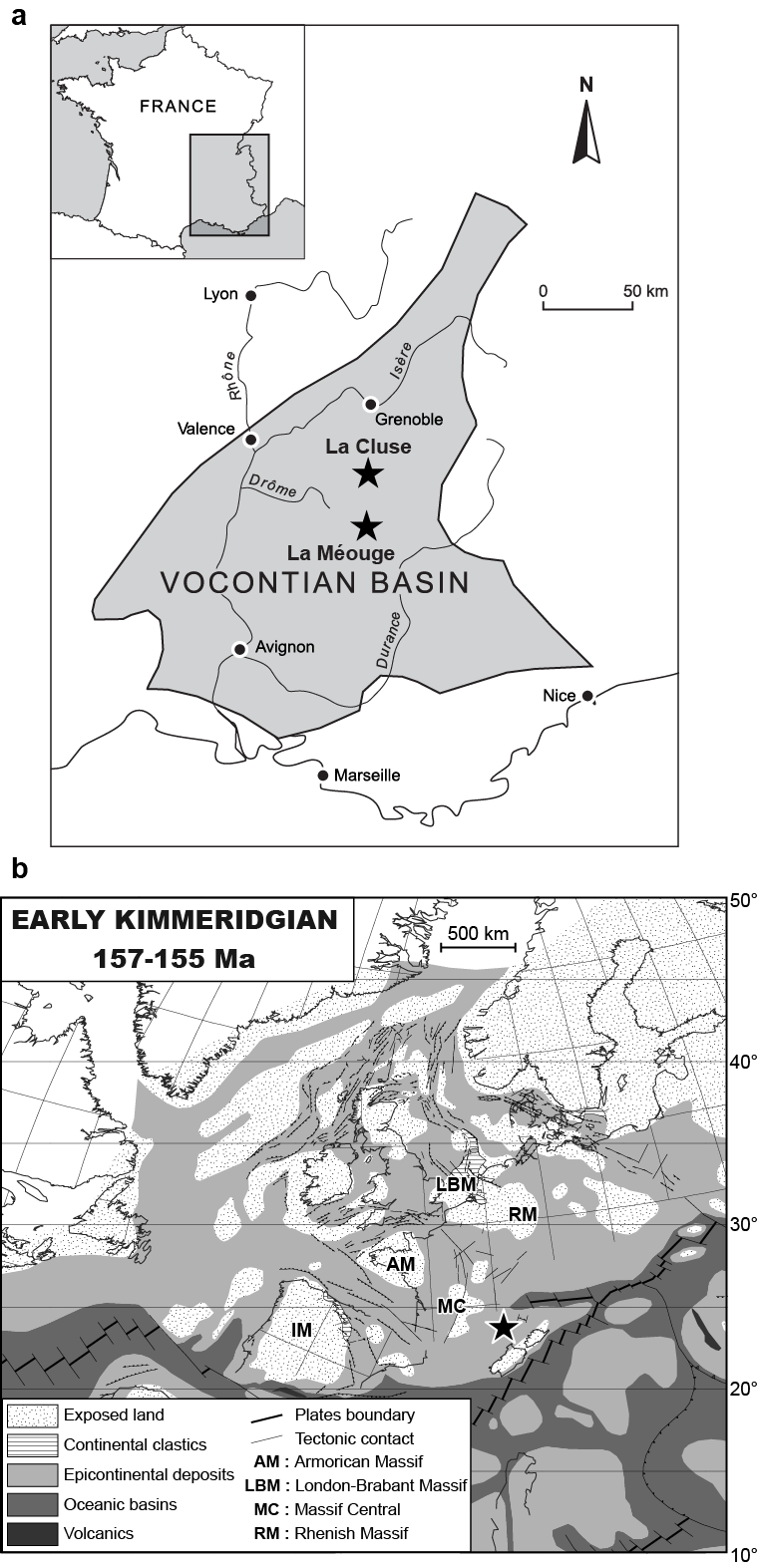


**Supplementary Figure S1:** **(a)** Present location of the La Cluse and the La Méouge sections in the Vocontian Basin of southeastern France (modified from Boulila et al.^13^). **(b)** Paleogeographic framework of the Vocontian Basin of southeastern France (indicated with the star) during the Early Kimmeridgian (modified from Thierry^86^). This figure was generated by Adobe Illustrator CS6 software (https://helpx.adobe.com/fr/illustrator/using/links-info.html).

**
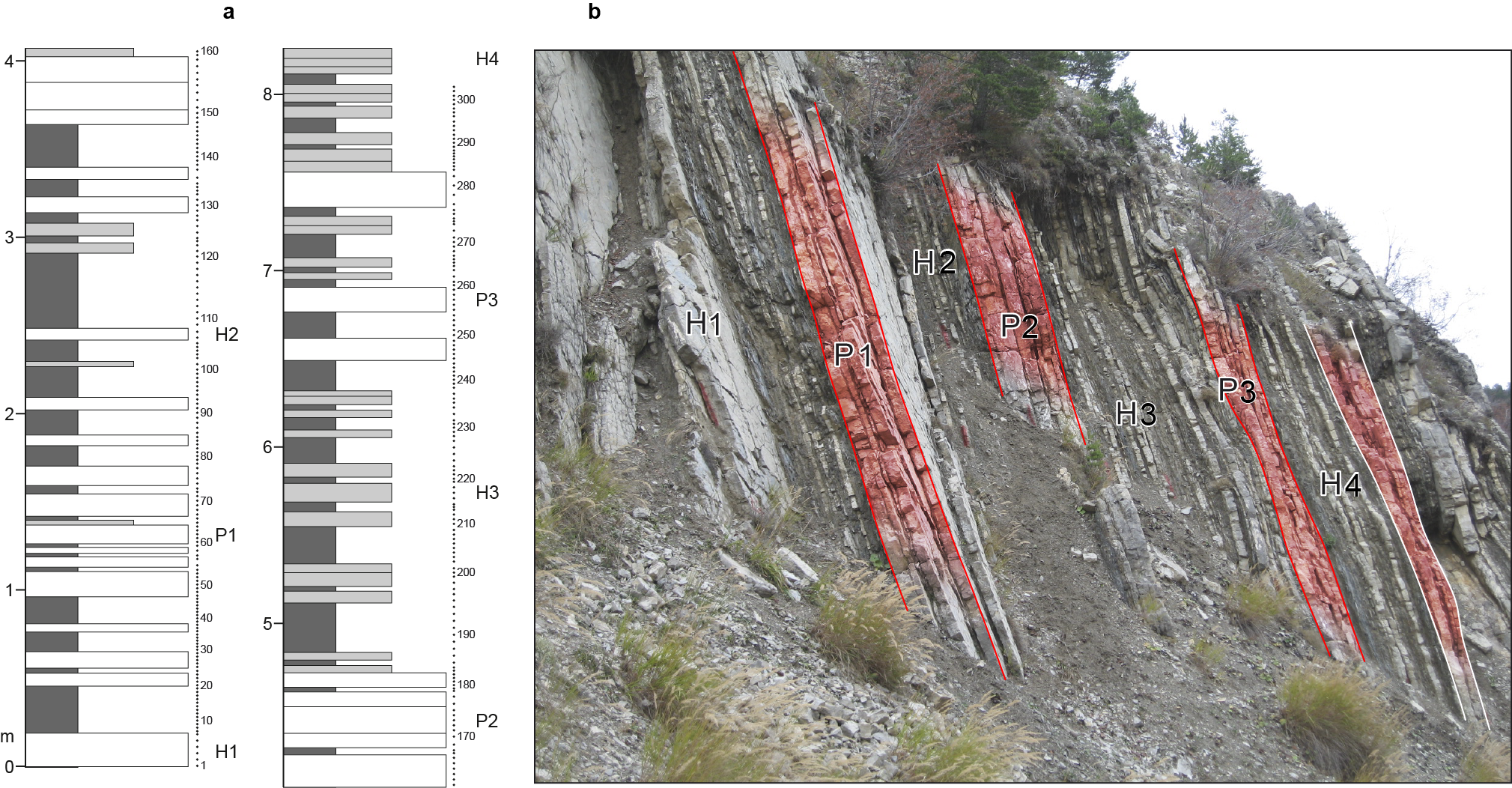
**

**Supplementary Figure S2:** Field photograph of the lower part of the studied La Cluse section. **(a)** Lithostratigraphic log, along with the collected samples. The log is from one section, but splitted into two parts in order to visualize the collected samples. **(b)** Photograph of the La Cluse outcrops showing elementary marl-limestone alternations (couplets) bundled (modulated) by precession (P) and half-precession (H) cycles. H1 through H4 are half-precession cycle boundaries. P9 through P3 are precession cycle boundaries. Precession and half-precession boundaries are expressed as massive or more resistant limestone beds (carbonate-rich intervals, see Fig. 2). The small, elementary couplets correspond to the 1,500 year cycle.


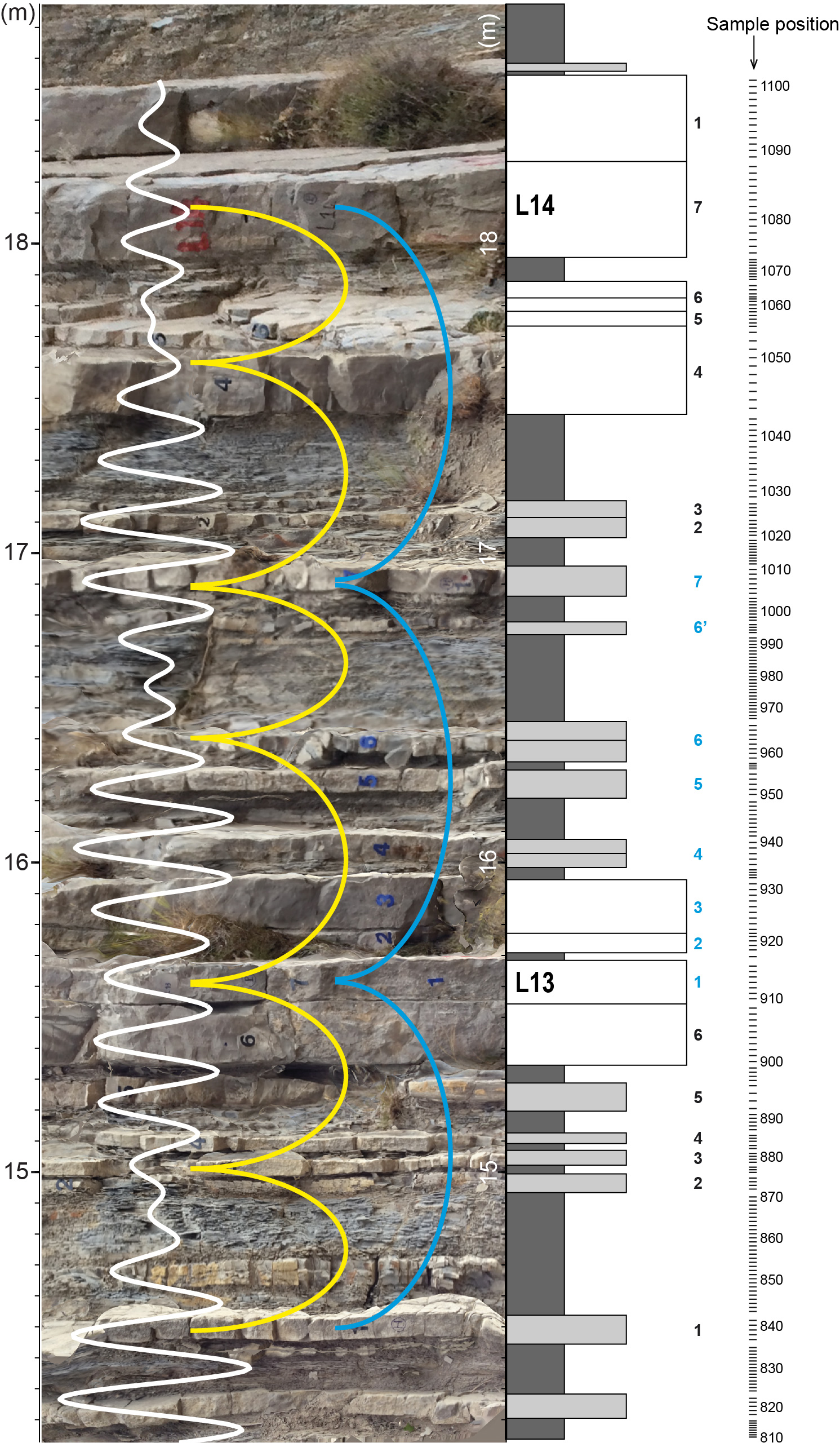


**Supplementary Figure S3:** Illutration of field interpretation of the 1.5 kyr cycle, bundled by quarter- and half-precession cycles along with the lithostratigraphic log.


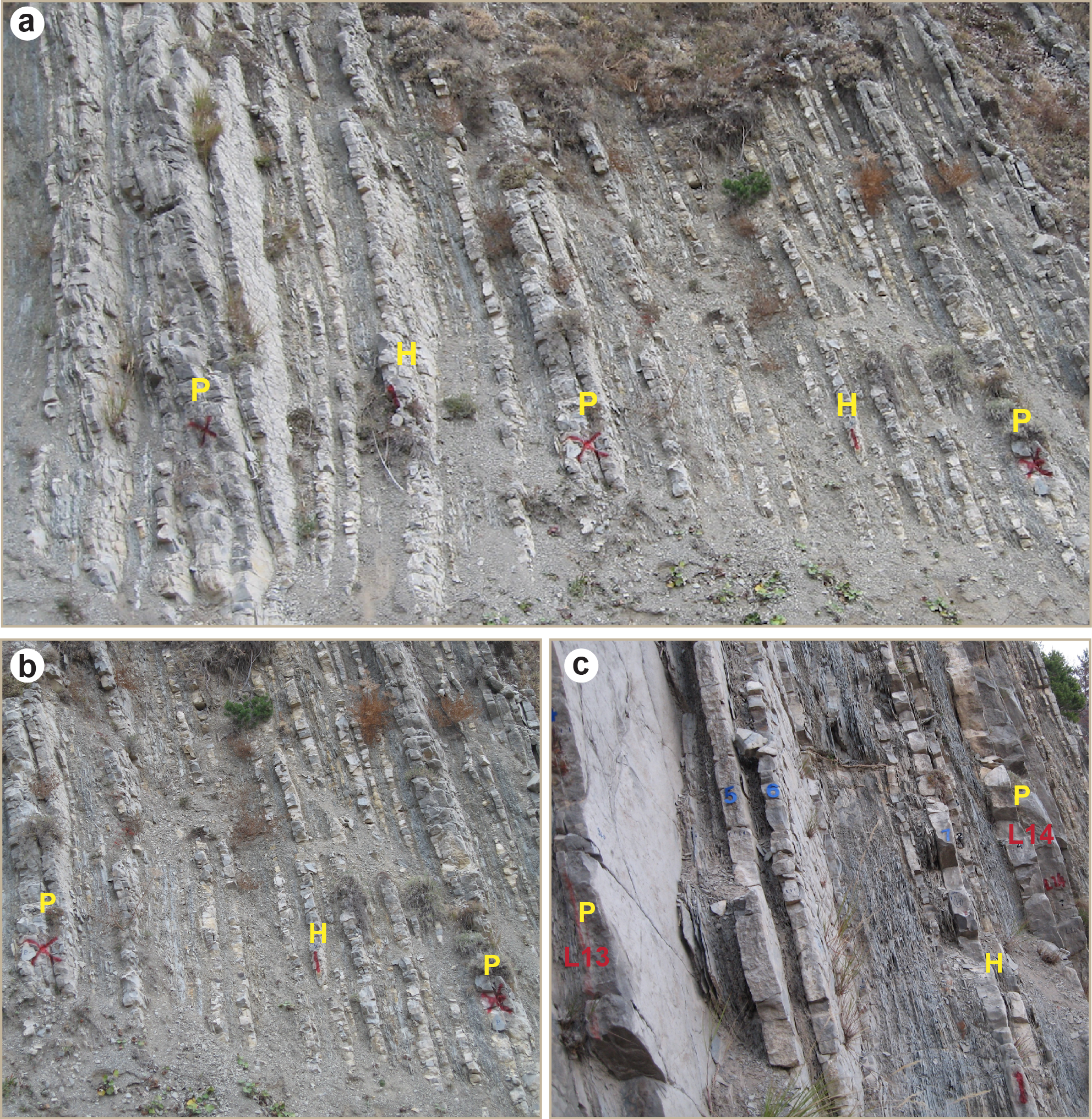


**Supplementary Figure S4:** Illutration of field interpretation of the 1.5 kyr cycle, bundled by half-precession (H) and precession (P) cycles. **(a)** A photograph covering two complete precession cycles. **(b)** A photograph covering one complete precession cycle. **(c)** A photograph showing the numbered DO equivalent lithological cycles within each half-precession cycle, interpreted during the field work (numbers are in blue or black labels).


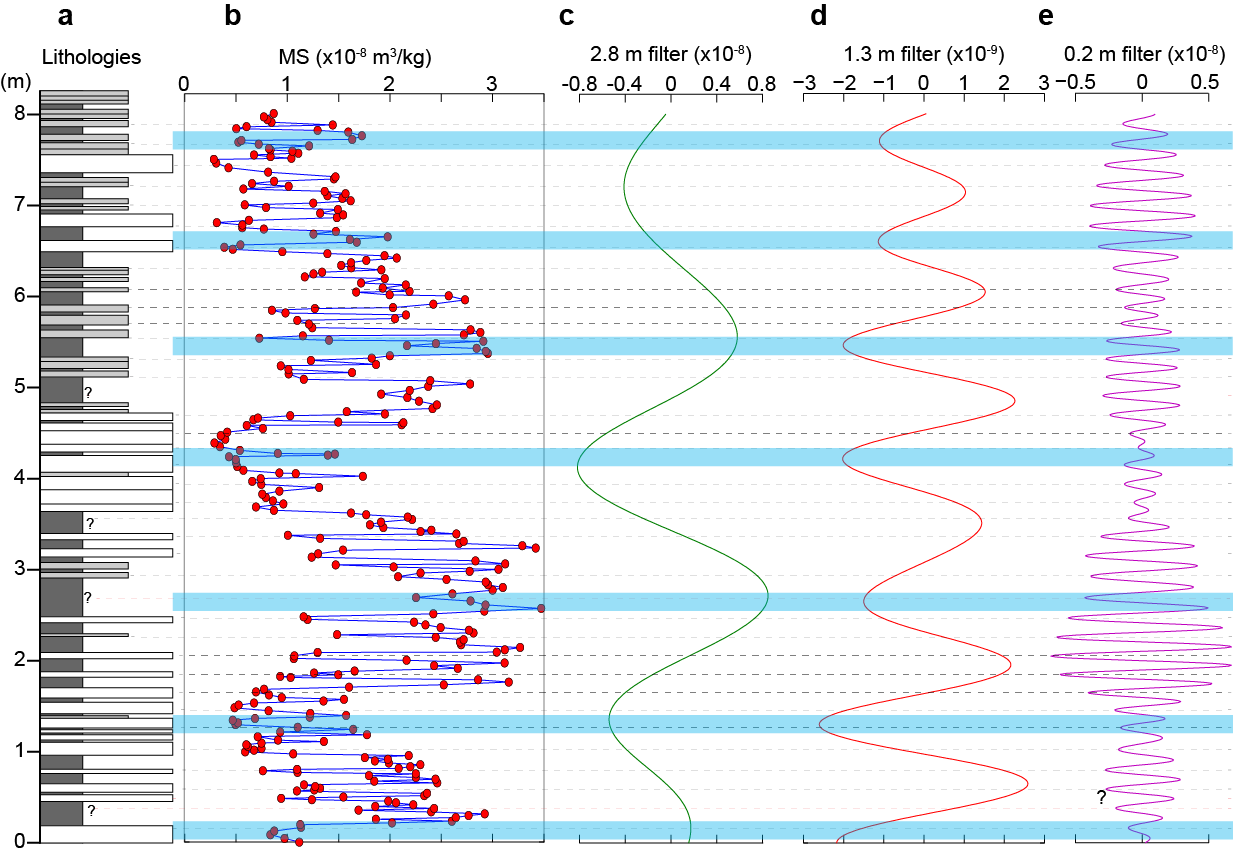


**Figure S5:** A focus on the lower part of the La Cluse section, to show the ratio of 1/12 to 1/15 between the presumed DO analogous and precession periodicities. **(a)** Lithostratigraphy of the lower part of the Lothari ammonite subzone of the Early Kimmeridgian (Late Jurassic, ~155 Ma). For lithology, white: limestone, light grey: marly limestone, and dark grey: marls. **(b)** MS data**.** Higher values in clay-rich and lower values in carbonate-rich sediments. **(c)** 2.8 m bandpass filtering of the precession related cycles. **(d)** 1.3 m bandpass filtering of the half-precession related cycles**. (e)** 0.2 m bandpass filtering of cycles matching the thinnest marl-limestone alternations or couplets (the 1.5 kyr analogous cycles, see Fig. S6 below), shown by horizontal dashed lines**.** Question marks in the lithological log indicate MS detected fine variations in the lithology, which were not highlighted from the outcrop description. Horizontal light-blue bars indicate half-precession equivalent cycles (see Fig. S6 below).


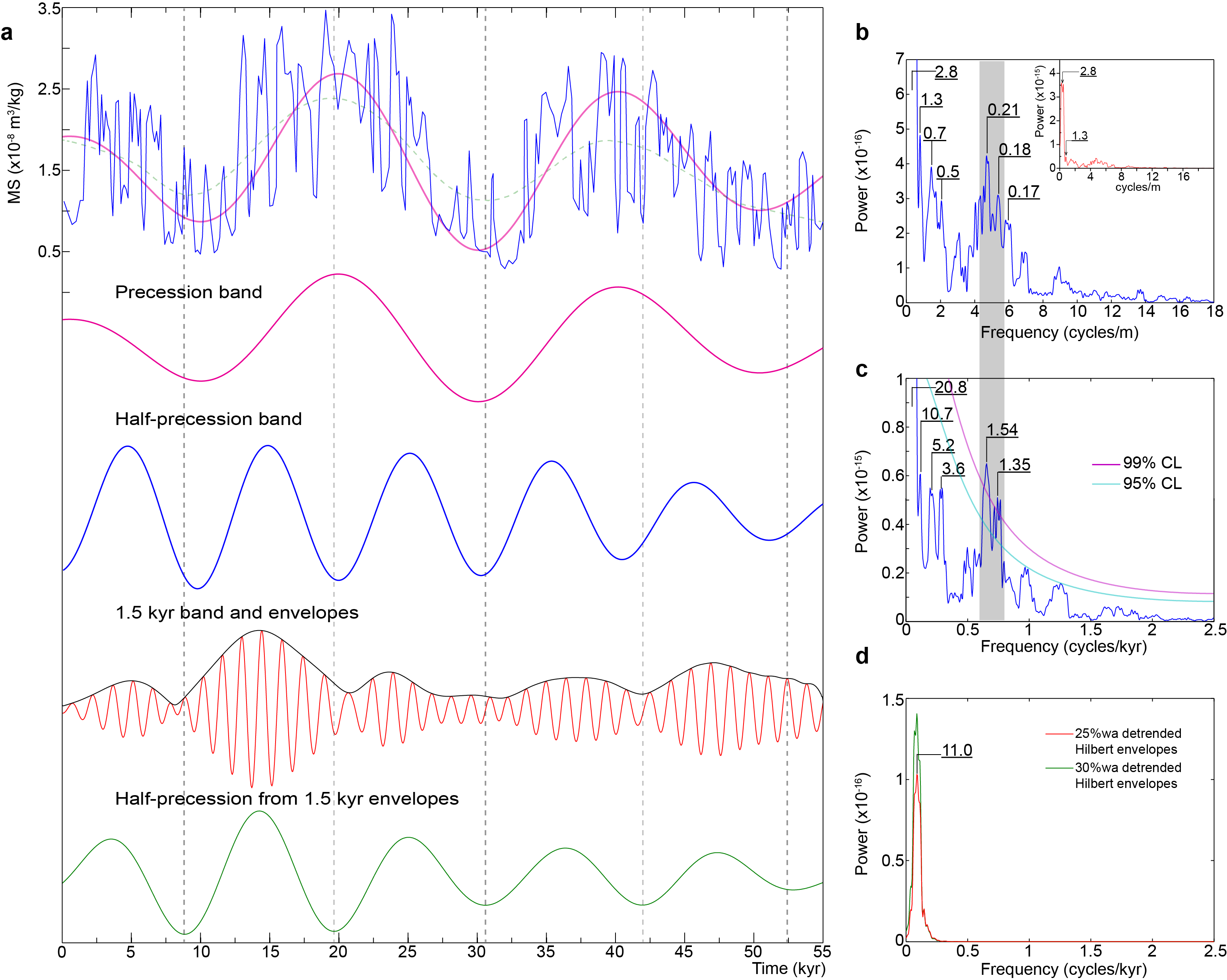


**Figure S6:** Time-series analysis of magnetic susceptibility (MS) proxy data of the lower part of the La Cluse section (the same interval as in Fig. S5, but in the time domain). **(a)** MS data in the time domain, along with 25% weighted average of the series and bandpass filtering at precession, half-precession and 1.5 kyr bands. Note that amplitude envelopes of the 1.5 kyr cycle band match the half-precession cycles. **(b)** Power spectra in the depth domain. **(c)** Power spectra in the time domain. **(d)** Power spectra of amplitude envelopes of the 1.5 kyr band in the depth domain. The power of the strongest peak of the precession in ‘b’ and ‘c’ is truncated in order to visualize higher frequency bands. For details on the whole spectrum, see the ‘inset’ in ‘b’. The values of periods of the precession and its harmonics, i.e. related peaks in ‘b’ and ‘c’ are further highlighted via GLS periodogram (Supplementary figure S7 below), and zero-padding.

**
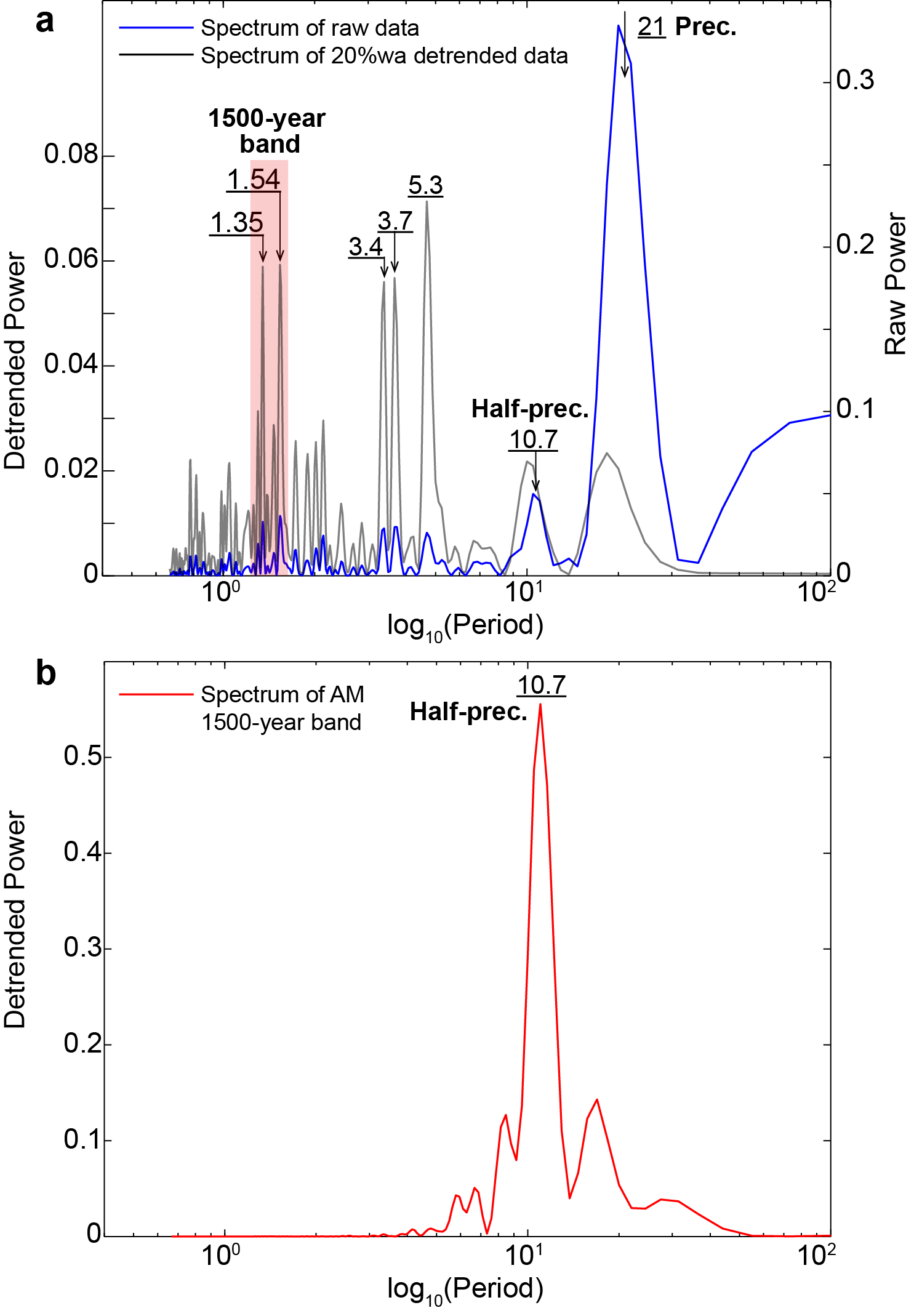
**

**Supplementary Figure S7:** Generalized Lomb-Scargle (GLS) periodograms of magnetic susceptibility (MS) proxy data of the lower part of the La Cluse section (the same interval as in Supplementary figure S5, but in the time domain). **(a)** GLS periodograms of the tuned MS data. **(b)** GLS periodogram of AM envelopes of the 1,500 year cycle band.


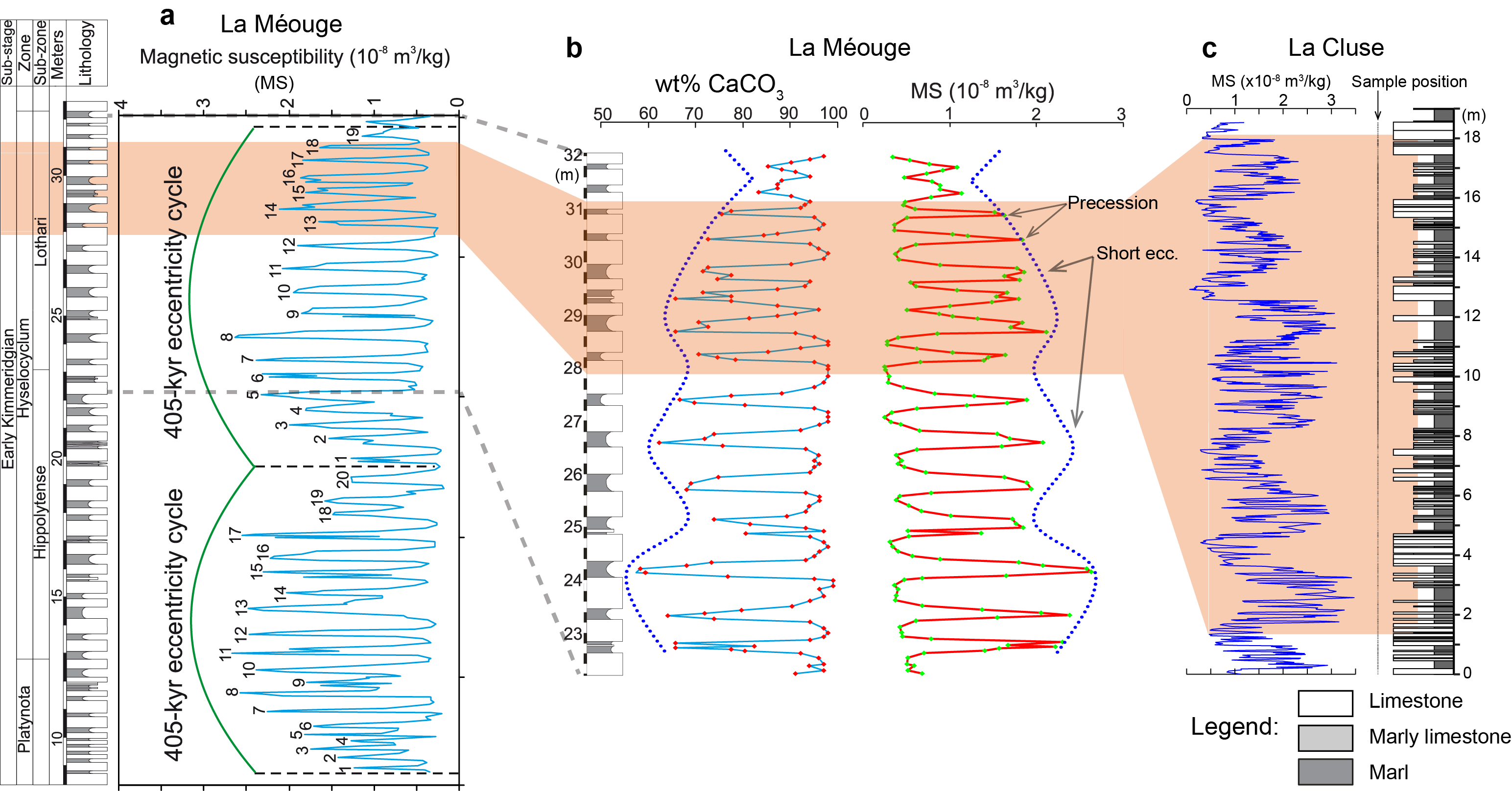


**Supplementary Figure S8:** Illustration of the approach used to convert thickness into time. **(a)** Cyclostratigraphy of the La Méouge sedimentary section from high-resolution magnetic susceptibility (MS) data^12,13^. The target 405 kyr cycles related to the long eccentricity are shown. Each 405 kyr cycle contains almost twenty marl-limestone couplets corresponding to the precession. **(b)** High-resolution MS and %CaCO_3_ data of the interval from ~22.2 to ~32 m in ‘a’. Note the strong negative correlation between MS and %CaCO_3_. Short eccentricity (100 kyr) cycles bundling (modulating) the couplets are also shown. Dots on curves indicate samples. **(c)** Lithostratigraphy and MS data of the studied La Cluse section. This interval match approximately six precession cycles (couplets) at the La Méouge^12,13^. The ~3.20 m thick interval at the La Méouge corresponds to the ~17 m thick interval at the La Cluse (orange-shaded interval). Note the extremely higher sedimentation rates at the La Cluse.


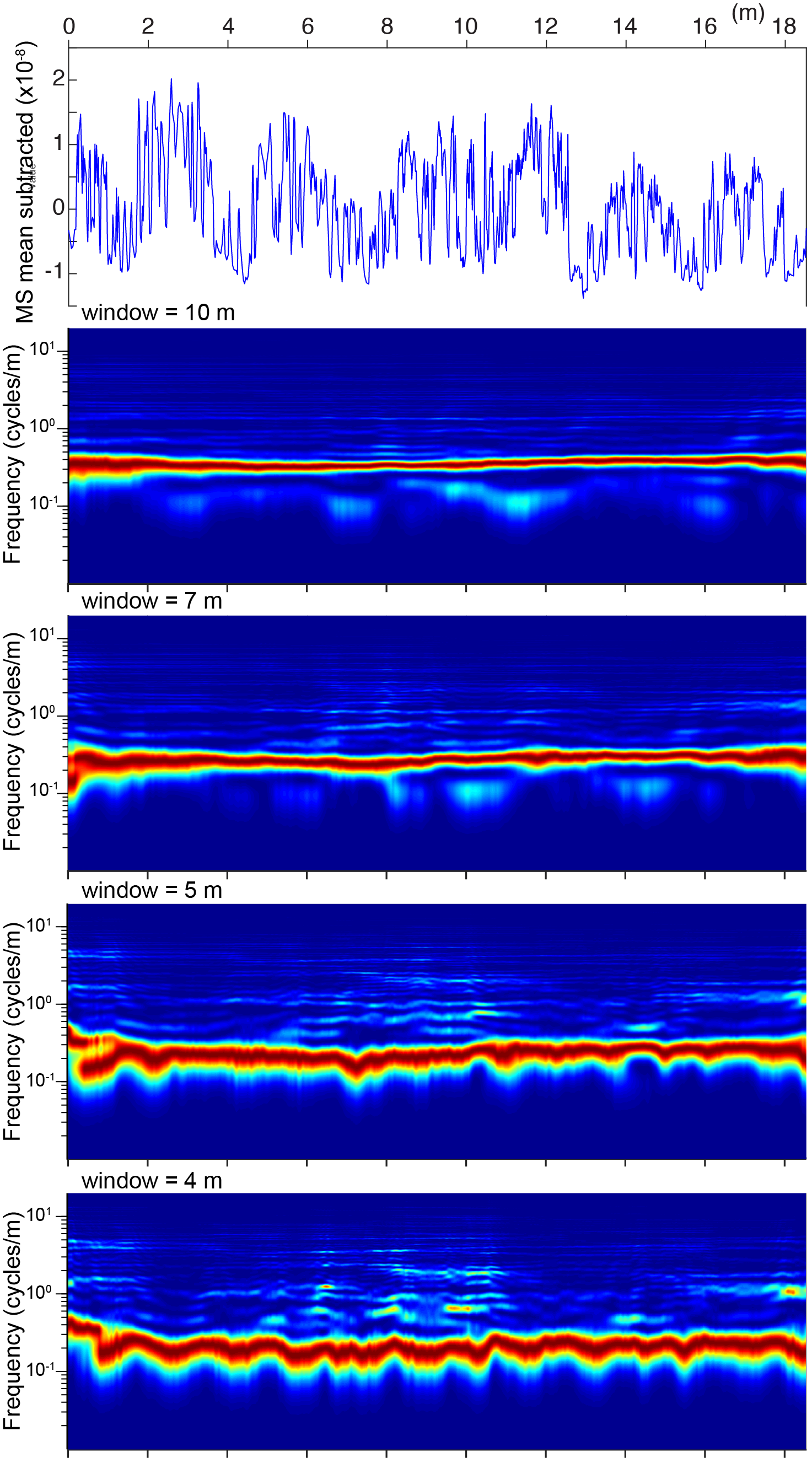


**Supplementary Figure S9:** Evolutive FFT spectrograms of the raw MS data in the stratigraphic domain using different sliding windows 10, 7, 5 and 4 m to capture the precession scale cyclicity. The strong spectral line depicting the precession wavelength does not show any disruption that may reflect potential hiatuses. Note the long trend upto section towards higher frequencies, reflecting a progressive decreasee in sedimentation rate.


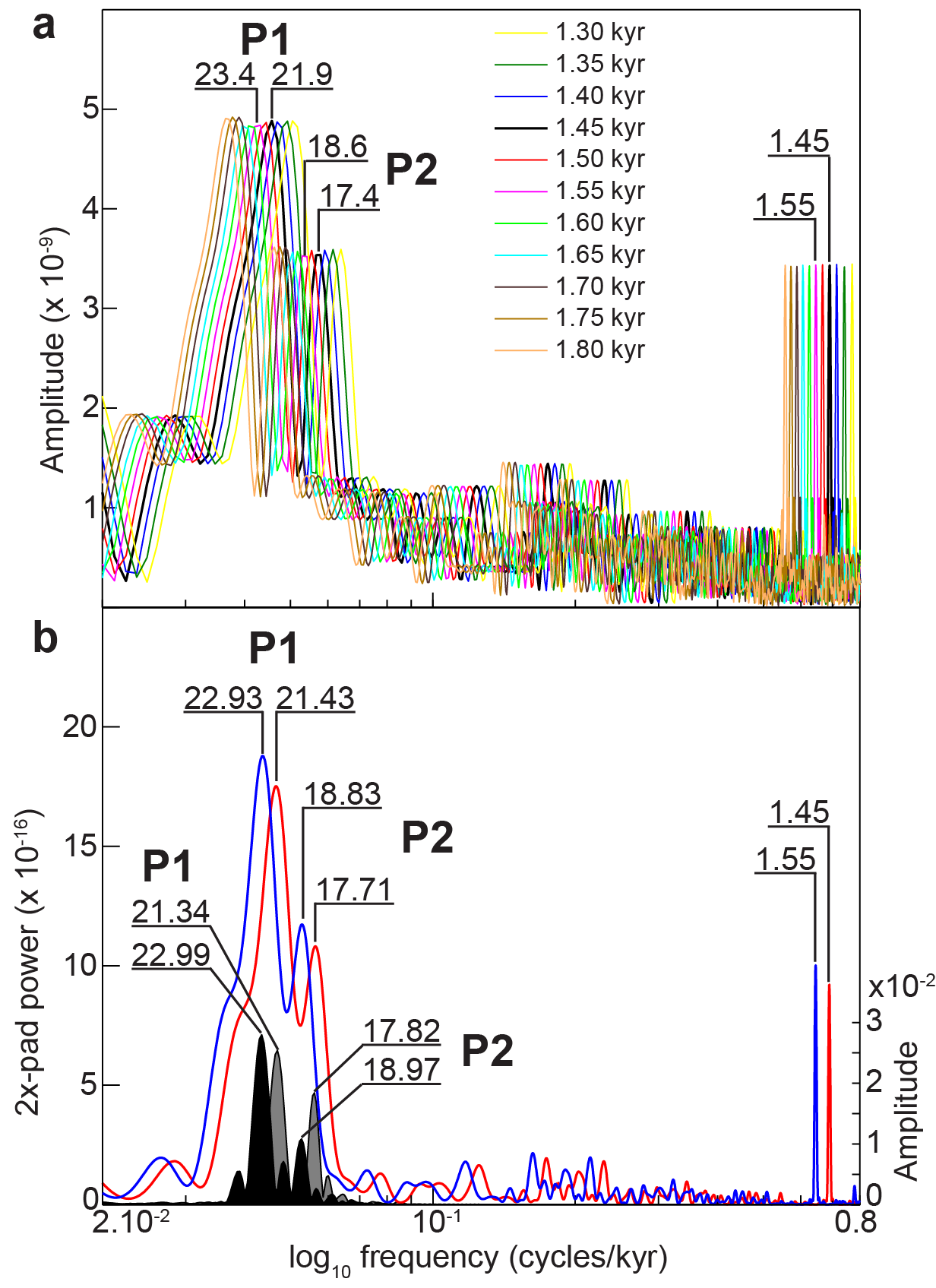


**Supplementary Figure S10:** 1.5 kyr tuning of wavelengths of elementary marl-limestone couplets, and comparison of the calibrated precession periods to the theoretically predicted Jurassic and present values^16^. **(a)** Spectra of 0.05 kyr incremental tuning. **(b)** Details on power spectra of 1.45 and 1.55 kyr tunings and amplitude spectra of La2004 precession of the intervals 0-420 ka (black) and 155.400-155.820 ka (grey). The length of these two intervals was fixed as three times long than the recovered sedimentary record. This latter was 3x-padded prior to spectral analysis to enhance the spectral resolution. P1 and P2 are the dominant precession cyclicities.


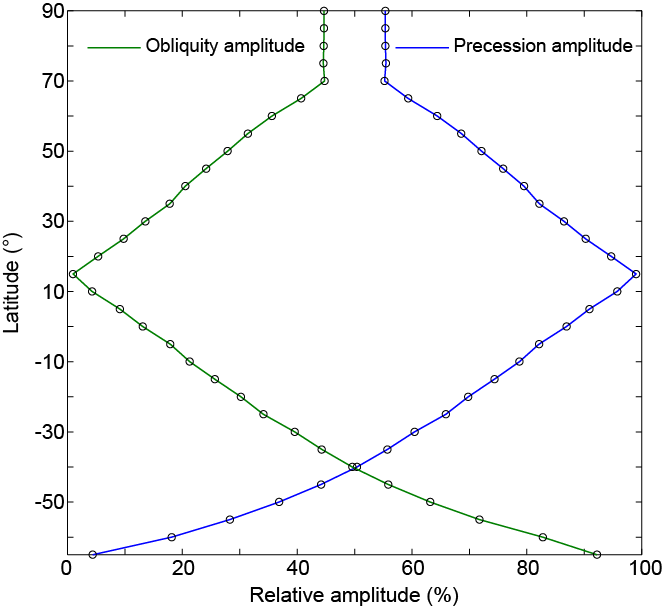


**Supplementary Figure S11:** Relative amplitudes of precession and obliquity as expressed in variations of the boreal summer insolation through latitudes (positive for north hemisphere, and negative for south hemisphere, latitude increment = 5°).

**
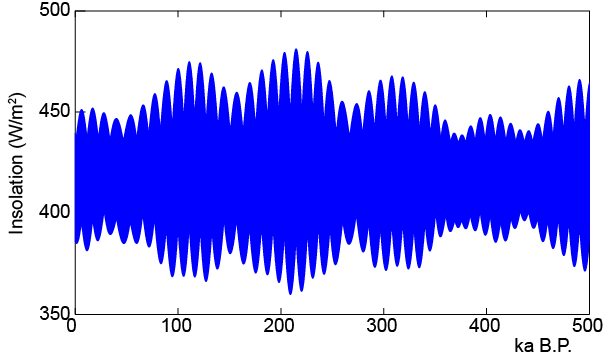
**

**Supplementary Figure S12:** Equatorial insolation over the past 500 ka, modelled with a sampling step of one month. The blue-shaded area depicts the annual cycle, which is modulated by the first precession harmonic (half-precession), the precession and the eccentricity cycles.


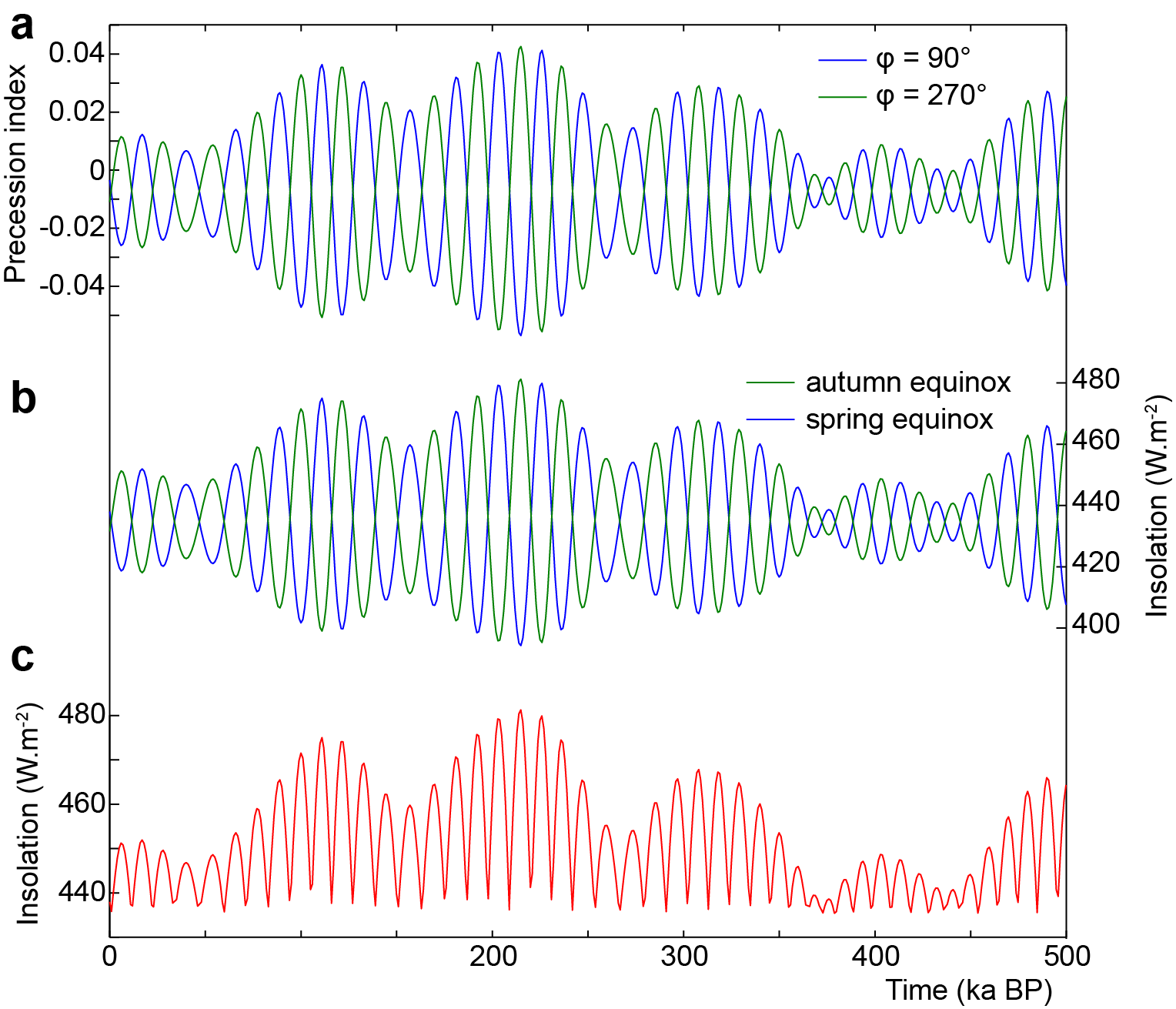


**Supplementary Figure S13:** Potential origin of the half-precession cycles at low latitudes. **(a)** Precession index with two phase φ angles from the vernal point. **(b)** Equatorial daily insolation at autumn and spring equinoxes. **(c)** Sum of larger values of autumn and spring daily insolation (truncated at mean values).


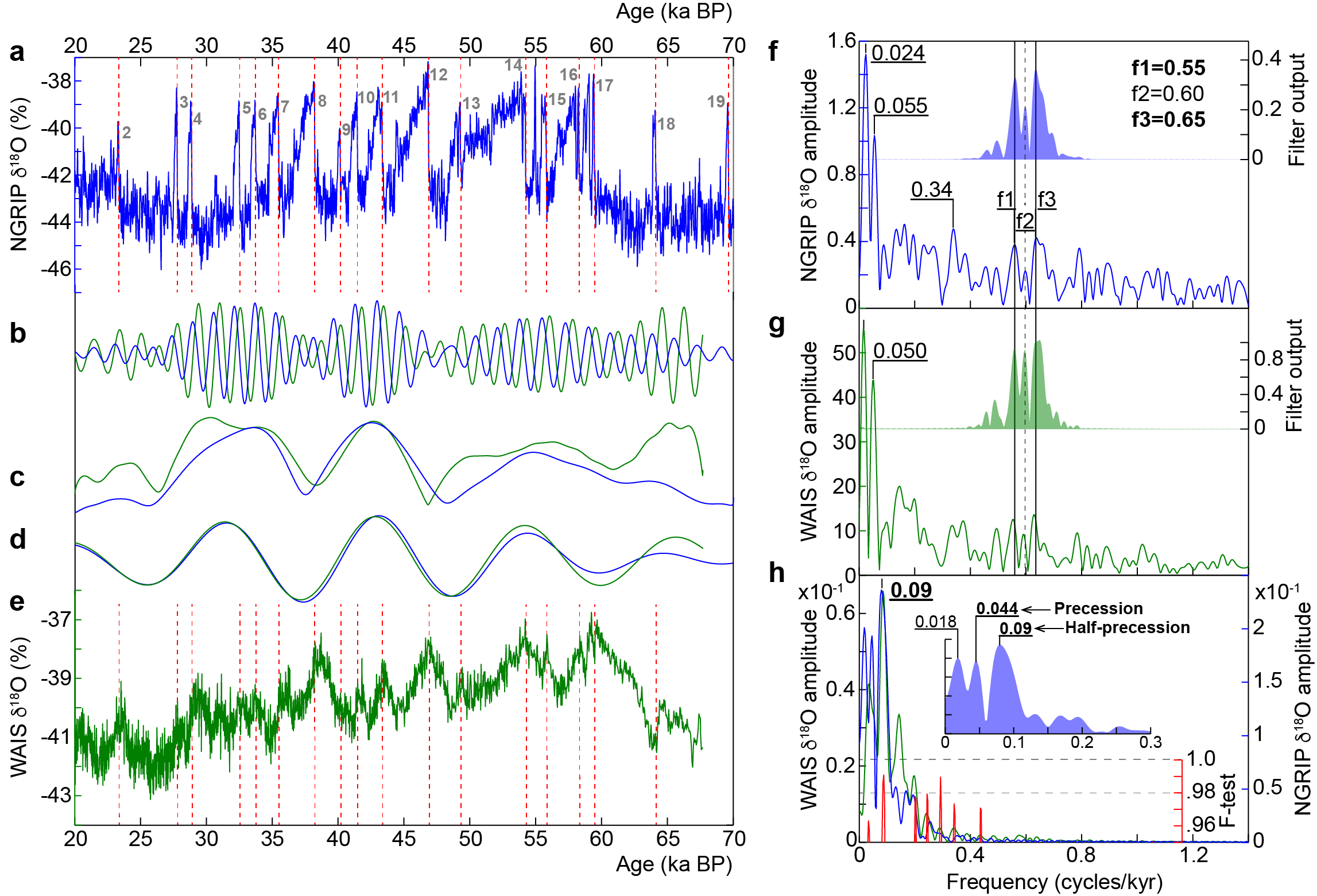


**Supplementary Figure S14:** Amplitude modulation (AM) analysis of δ^18^O NGRIP and WAIS data at the 1.5 kyr cycle band within the interval 20-70 ka, where much of 1.5 kyr power and DO cycles are focused (see main Fig. 7b). **(a)** δ^18^O NGRIP data (references as in main Fig. 7a). **(b)** Bandpass flitered δ^18^O data at the 1.5 kyr cycle band (0.6 ±0.1 cycles/kyr) (NGRIP in blue and WAIS in green). **(c)** Extracted AM envelopes of the 1.5 kyr cycle band via Hilbert transform (same color codes for NGRIP and WAIS). **(d)** 10 kyr bandpass filered (0.095 ±0.02 cycles/kyr) AM envelopes. **(e)** Antarctic δ^18^O data from the WAIS ice core^95^ using WD2014 chronology^96^. Vertical red lines indicate the DO events. **(f)** NGRIP amplitude spectra of raw δ^18^O data and filter output (*inset*) showing the three beating frequencies (f1, f2 and f3). f1 and f3 are indicated in bold text because their respective cycle amplitudes are higher than f2 related cycle. **(g)** WAIS amplitude spectra of raw δ^18^O data and filter output (*inset*). f1, f2 and f3 are slightly shifted towards lower frequencies, a shift within spectral bandwidth resolution and agescale uncertainties **(h)** Amplitude spectra of AM envelopes of the 1.5 kyr cycle band (NGRIP in blue and WAIS in green) along with the F-test values for NGRIP. *Inset*: The same NGRIP amplitude spectrum but over a shorter frequency axis (0 to 0.3 cycles/kyr) to highlight resulting AM cycles of precession and half-precession (shown).


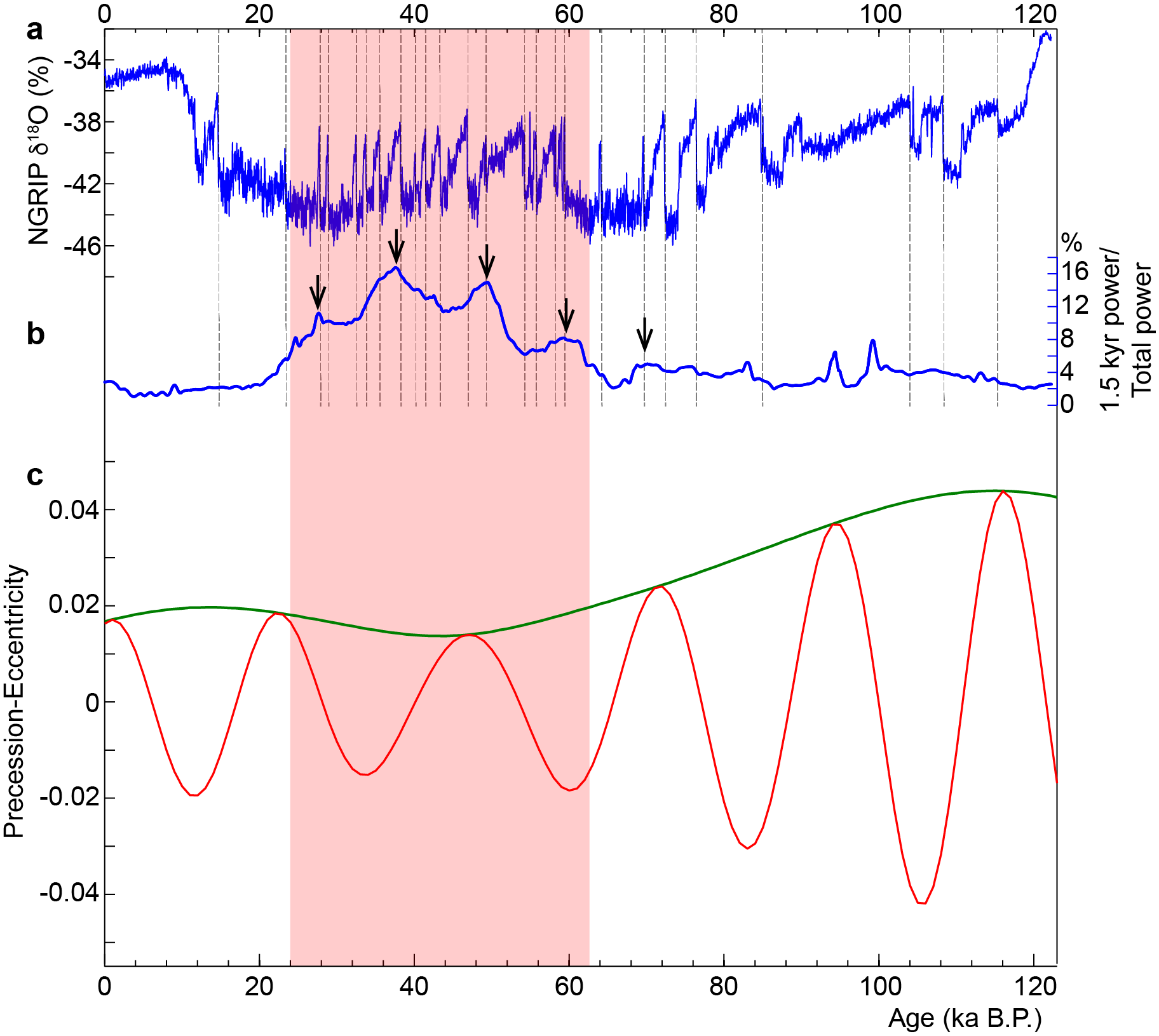


**Supplementary Figure S15:** Correlation between DO events and precession-eccentricity variations. **‘a’** and **‘b’** as in main Fig. 7a,b. **(c)** Precession and eccentricity variations. Orange-shaded area depicts the maximal power of DO events correlated to low values of orbital eccentricity variations.
